# Tools to kill: Genome of one of the most destructive plant pathogenic fungi *Macrophomina phaseolina*

**DOI:** 10.1186/1471-2164-13-493

**Published:** 2012-09-19

**Authors:** Md Shahidul Islam, Md Samiul Haque, Mohammad Moinul Islam, Emdadul Mannan Emdad, Abdul Halim, Quazi Md Mosaddeque Hossen, Md Zakir Hossain, Borhan Ahmed, Sifatur Rahim, Md Sharifur Rahman, Md Monjurul Alam, Shaobin Hou, Xuehua Wan, Jennifer A Saito, Maqsudul Alam

**Affiliations:** 1 Basic and Applied Research on Jute Project, Bangladesh Jute Research Institute, Manik Mia Avenue, Dhaka 1207, Bangladesh; 2 Advanced Studies in Genomics, Proteomics and Bioinformatics, University of Hawaii, 2565 McCarthy Mall, Keller 319, Honolulu, Hawaii 96822, USA; 3 Centre for Chemical Biology, Universiti Sains Malaysia, Penang, 11800, Malaysia

**Keywords:** Genome sequencing, Phytopathogens, Charcoal rot, Phenotypic microarray

## Abstract

**Background:**

*Macrophomina phaseolina* is one of the most destructive necrotrophic fungal pathogens that infect more than 500 plant species throughout the world. It can grow rapidly in infected plants and subsequently produces a large amount of sclerotia that plugs the vessels, resulting in wilting of the plant.

**Results:**

We sequenced and assembled ~49 Mb into 15 super-scaffolds covering 92.83% of the *M. phaseolina* genome. We predict 14,249 open reading frames (ORFs) of which 9,934 are validated by the transcriptome. This phytopathogen has an abundance of secreted oxidases, peroxidases, and hydrolytic enzymes for degrading cell wall polysaccharides and lignocelluloses to penetrate into the host tissue. To overcome the host plant defense response, *M. phaseolina* encodes a significant number of P450s, MFS type membrane transporters, glycosidases, transposases, and secondary metabolites in comparison to all sequenced ascomycete species. A strikingly distinct set of carbohydrate esterases (CE) are present in *M. phaseolina*, with the CE9 and CE10 families remarkably higher than any other fungi. The phenotypic microarray data indicates that *M. phaseolina* can adapt to a wide range of osmotic and pH environments. As a broad host range pathogen, *M. phaseolina* possesses a large number of pathogen-host interaction genes including those for adhesion, signal transduction, cell wall breakdown, purine biosynthesis, and potent mycotoxin patulin.

**Conclusions:**

The *M. phaseolina* genome provides a framework of the infection process at the cytological and molecular level which uses a diverse arsenal of enzymatic and toxin tools to destroy the host plants. Further understanding of the *M. phaseolina* genome-based plant-pathogen interactions will be instrumental in designing rational strategies for disease control, essential to ensuring global agricultural crop production and security.

## Background

*Macrophomina phaseolina,* a global devastating necrotrophic fungal pathogen, infects more than 500 plant hosts [1]. It includes major food crops (maize, sorghum [2]), pulse crops (common bean [3], green gram [4]), fiber crops (jute [5], cotton [6]), and oil crops (soybean [1], sunflower [7], sesame [8]). Despite its wide host range, *Macrophomina* is a monotypic genus [9].

Diseases caused by *M. phaseolina* (e.g., seedling blight, charcoal rot, stem rot, and root rot) are favored with higher temperatures (30-35°C) and low soil moisture [10]. It is difficult to control *M. phaseolina* due to its persistence as sclerotia in the soil and plant debris [11]. Recently, increased incidence of the pathogen on diverse crop species has been reported worldwide [12-14], highlighting the importance of this disease to crop production in drought prone regions.

The fungus has a wide geographical distribution, and is especially found in tropical and subtropical countries with arid to semi-arid climates in Africa, Asia, Europe, and North and South America [15-17]. This pathogen can result in severe crop losses. For example, charcoal rot is a serious problem of soybean, which accounted for a total yield loss of $173.80 million in the United States during 2002 [18]. In Bangladesh, the fiber yield of jute is reduced by 30% due to this pathogen.

**Figure 1:**
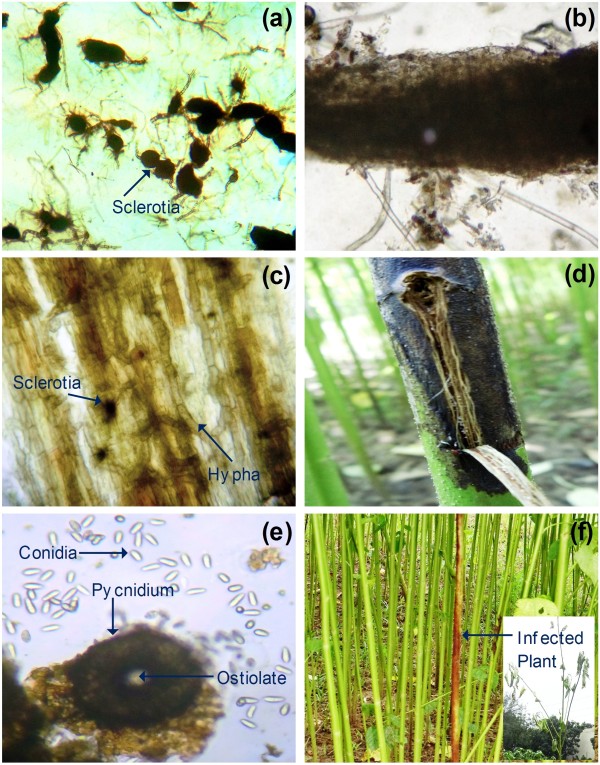
**Infection of jute by *M. phaseolina*. **(**a**) Stereomicrograph of sclerotia that exists in soil and crop residue. (**b**) Pathogen produces extensive and profuse aerial hyphae to invade the stem bark. (**c**) Longitudinal section of stem bark showing inter- and intracellular mycelium and sclerotia. (**d**) During early rainy season, hyphae penetrate the plant cell wall and produce disease symptoms. (**e**) Light micrograph of globose ostiolate pycnidia and spores of *M. phaseolina*. (**f**) Diseased plants showing infection of the stem, which eventually wilt and prematurely die (Inset).

*M. phaseolina* is an anamorphic fungus in the ascomycete family Botryosphaeriaceae. The fungus can remain viable for more than 4 years in soil and crop residue as sclerotia (Figure 1a) [11]. The *M. phaseolina* hyphae initially invade the cortical tissue of jute plants, followed by sclerotia formation, causing stem rot disease (Figure 1b, c). Gray-black mycelia and sclerotia are produced (Figure 1c) and the infected area exhibits disease symptoms (Figure 1d). The conidia are hyaline, aseptate, thin-walled, and elliptical (Figure 1e). Under favorable conditions, hyphae germinate from the sclerotia and infect the roots of the host plant by penetrating the plant cell wall through mechanical pressure and/or chemical softening [19]. The disease progresses from leaf yellowing to wilting and ultimately plant death (Figure 1f). 

Currently, genetic information on *M. phaseolina* is scarce with only 176 expressed sequence tags (ESTs) and 903 nucleotide sequences in the National Center for Biotechnology Information (NCBI). Here we report the draft genome sequence of highly destructive plant pathogen *M. phaseolina* to gain insight into the molecular basis of pathogenesis.

## Results and discussion

### Genome sequencing and assembly

The genome of *M. phaseolina* was sequenced using a whole-genome shotgun approach. A total of 6.92 Gb of raw sequence was generated from a combination of 454 and Illumina platforms (Additional file 1: Table S1). The resulting assembly is 49.29 Mb of which 98.53% is non-gapped sequence (Table 1; Additional file 1: Table S2). Mapping with Newbler GS Reference Mapper (v2.5.3) showed 96.50% reads and 99.11% bases mapped to the reference assembly. The draft genome sequence consists of 94 scaffolds, with 15 super scaffolds covering 92.83% of the total assembled length (Additional file 1: Table S2). We predicted 14,249 protein-coding genes and 9,934 were validated by the transcriptome ( Additional file 1: Table S3).

**Table 1:** Genome assembly and annotation statistics

**Genome features**	
Strain	MS6
Sequence coverage (fold)	13
Genome Size (Mb)	49.295
Total scaffolds	94
No. of scaffolds (≥ 1 Mb)	15
N50 scaffold length (Mb)	3.39
Number of N50 scaffolds	6
Number of genes	14,249
No. of genes in 15 scaffolds (≥ 1 Mb)	14,071
Number of genes found in cDNA	9,934
Median gene length (bp)	1,265
Repetitive sequence (%)	2.84
Transposable elements (%)	3.98
NCBI accession	AHHD00000000

**Figure 2:**
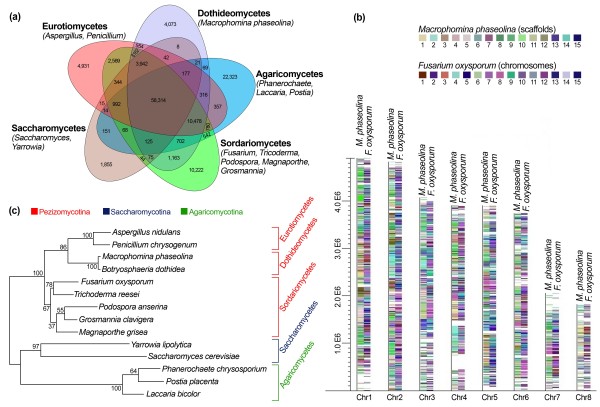
**Homology, syntenic, and phylogenetic relationship of *M. phaseolina*. **(**a**) Comparative analyses of orthologous and paralogous gene families of 13 fungal species. Number of genes are presented for each component. Clustering was done by using OrthoMCL (MCL-10-201). (**b**) Synteny of *M. phaseolina* and *Fusarium oxysporum* using whole genome data. The reference genome (*Aspergillus fumigatus*) is broken up into eight chromosomes and syntenic regions are represented by two vertical columns with color. (**c**) Phylogenetic tree showing the positioning of *M. phaseolina* within the pezizomycotina.

We examined the homology between *M. phaseolina* and 12 other fungal genomes under the classes of Saccharomycetes, Sordariomycetes, Agaricomycetes, and Eurotiomycetes. The results revealed that 71% of the genes in the *M. phaseolina* genome have homologs in other fungal genomes and the remaining 29% are orphan genes (Figure 2a). Among the orphan genes, 51.11% are found in the transcriptome.

The comparison of *M. phaseolina* protein families with other ascomycete fungal species is shown in Table 2 (also see Additional file 1: Table S4). The genome contains 13.07% (1,863) secreted proteins as compared to 7-10% in other plant pathogens [20]. Surprisingly, *M. phaseolina* has the lowest number of proteases among ascomycete fungal species [21] (Table 2). On the other hand, the genome is distinct from other fungi by having the highest number of cytochrome P450, glycosidase, and secondary metabolite backbone genes. We predict that this might be one of the main strategies of *M. phaseolina* to overcome the host plant defense response by using various secondary metabolites. 

**Table 2:** **Sizes of selected protein families in * M. phaseolina* and other fungi**

**Protein family**^**a**^	**MP**^**b**^	**FO**	**FG**	**MO**	**BC**	**SS**	**NC**	**AN**	**AF**	**PCH**	**PP**
Fungal specific transcription factors	156	101	192	95	118	90	89	209	169	65	63
C2H2 zinc finger transcription factors	66	73	85	58	48	54	63	58	51	77	42
Zn2/Cys6 transcription factors	113	370	376	155	142	108	110	307	230	146	118
Major facilitator superfamily	270	352	274	198	225	167	110	279	232	141	184
Cytochrome P450	256	178	112	137	129	93	40	116	74	155	236
Pth11-like G-protein coupled receptor	44	55	51	60	22	23	28	39	15	14	26
Protein kinases	140	160	129	129	124	164	111	127	131	106	56
Histidine kinase	1	37	20	6	3	5	8	12	6	19	24
Heterokaryon incompatibility	65	82	88	41	59	34	45	7	8	3	2
Serine proteases	1	12	60/150^c^	56/91	19/34	20/33	32/74	53/136	29/46	0	2
Subtilisin	19	36	16/24	26/29	4/7	4/6	6/10	3/4	3/7	12	33
Trypsin	2	3	2/3	3/3	1/1	1/1	0/2	1/2	0/0	0	0
Carboxypeptidase	19	31	12/21	7/8	7/9	8/ 11	6/9	5/12	14/15	24	22
Aspartic protease	4	0	15/18	14/19	11/14	9/21	15/19	7/16	7/9	38	18
Threonine protease	0	0	3/18	2/18	2/13	2/13	2/20	0/20	1/17	0	0
Cysteine protease	3	0	5/57	4/31	3/24	1/27	4/41	6/57	3/31	0	0
Metalloprotease	8	26	32/111	38/91	6/50	7/48	21/81	22/105	20/77	0	0
All proteases	113	261	354	250	135	142	235	334	180	228	325
Lipase	53	61	4/31	2/23	3/28	2/25	0/16	2/27	3/25	23	40
Esterase/thioesterase	108	95	70	64	70	58	42	63	52	74	69
Glycoside hydrolase related	219	168	159	198	120	126	137	200	165	180	144
Transposases	101	19	17	15	73	426	15	15	109	12	11
Cutinase	10	12	12	18	11	8	3	4	5	0	0
Polysaccharide lyase	16	23	25	9	25	20	5	24	27	4	6
Secondary metabolite backbone genes	75	34	37	32	37	29	15	58	40	51	39

^a^Corresponding InterPro codes are listed in Additional file 1: Table S4.

^b ^Fungal species are MP, *Macrophomina phaseolina*; FO, *Fusarium oxysporum;* FG, *Fusarium graminearum*; MO, *Magnaporthe oryzae*; BC, *Botrytis cinerea*; SS, *Sclerotinia sclerotiorum*; NC, *Neurospora crassa*; AN, *Aspergillus nidulans*; AF, *A. fumigatus*; PCH, *Phanerochaete chrysosporium,* and PP, *Postia placenta.*

^c^Fractions indicate the number of total proteins in each family that are secreted.

### Conserved syntenic and phylogenetic relationship

Pairwise comparison revealed that the genome structures of *M. phaseolina* and *Fusarium oxysporum*, one of the most important phytopathogenic and toxigenic fungi, have large areas of synteny (Figure 2b). Among the 14,249 *M. phaseolina* genes, 7,767 (54.10%) are shared with *F. oxysporum* (Additional file 2: Figure S1). The large number of shared genes may reflect common strategies for infecting a remarkably broad host range. Ninety seven percent of the *M. phaseolina* genome comprises non-repetitive sequences, and the orthologs shared with the *F. oxysporum* genome display an average 52% identity. A phylogenetic analysis revealed the evolutionary relationship among fungal taxa and the positioning of *M. phaseolina* within the pezizomycotina (Figure 2c).

### Global paralog network

Of the 14,249 predicted proteins, we found homologs for 7,999 proteins in nine fungal genomes. To characterize functional protein families in the *M. phaseolina* genome, we constructed a database in Pathway Studio (Ariadne Genomics Inc.) containing *M. phaseolina* proteins with functional annotation from the orthologs in nine other fungal genomes, predicted interologs, predicted pathways, and paralog network. The largest paralog families were identified by clustering the paralog network consisting of 6,210 paralog links representing paralog pairs with sequence homology above 30%. We identified 77 paralog families having more than six proteins (Additional file 2: Figure S2). The larger paralog families were the cytochrome P450 (151), MFS type membrane transporters (222), and transposases (101). The large number of transposons in the *M. phaseolina* genome suggests that they could be the primary mechanism for mutagenesis and gene duplications, which in turn may promote the ability of *M. phaseolina* to infect new plant species.

There was also a high number of paralogs for the oxido-reductase class of enzymes which includes dehydrogenases, aldehyde dehydrogenases, choline dehydrogenases, cytochrome P450 and aldose reductases. These oxido-reductase enzymes produce and utilize a large variety of secondary metabolites [22] which may trigger *M. phaseolina* to survive in a wide range of physical environments and facilitate to infect new plant species. The PTH11 paralog family consists of a large number of G-protein coupled receptors (GPCR; 44), which contain a cysteine-rich fungal extracellular membrane domain. This may facilitate *M. phaseolina* to be more virulent.

### Repetitive DNA and transposable elements

Wide virulence capabilities of a genome are often associated with transposon-mediated inactivation or deletion of pathogen-associated molecular pattern (PAMP)-encoding genes whose products trigger the plant adaptive immune system [23]. The *M. phaseolina* genome comprises 2.84% repetitive DNA and 3.98% transposable elements. The transposable elements are classified into 11 families (Table 3). Most of them are DNA transposases, with particular abundance of the subclasses gypsy (918), Ty1_Copia (331), and DDE_1 (242). LINE (184) and hAT (136) are also relatively abundant*.* Transposable elements appear to be tightly clustered in the genome (Additional file 2: Figure S3). Evidence for repeat-induced point mutation (RIP) within the transposable elements was searched using RIPCAL [24], but this mutational bias was not observed in the *M. phaseolina* genome (RIP index 0.87). The genome contains more fragmented pseudogenes (894) than processed pseudogenes (31) caused by mobile elements, but lacks duplicated pseudogenes. This is consistent with transposons making a greater contribution to genetic instability in *M. phaseolina.*

**Table 3:** **Families of transposable elements in the * M. phaseolina* genome**

**Family name**	**Class**	**Number**
LTR roo	Class I	4
DDE_1	Class I	242
**gypsy**	Class I	918
Ty1-Copia	Class I	331
LINE	Class I	184
hAT	Class II	136
helitron	Class II	15
cacta	Class II	4
Mariner	Class II	76
MuDR_A_B	Class II	57
piggybac	Class II	9
**Total**		**1976**

### Carbohydrate degrading enzymes

Phytopathogenic fungi secrete a cocktail of hydrolytic enzymes (including carbohydrate-active enzymes; CAZymes) for degrading the plant cell wall and penetrating into the host tissue [25]. The *M. phaseolina* genome encodes 362 putative CAZymes including 219 glycoside hydrolases (GH), 56 glycosyltransferases (GT), 65 carbohydrate esterases (CE), 6 carbohydrate binding modules (CBM), and 16 polysaccharide lyases (PL) comprising more than 80 distinct families. These enzymes do not appear to be tightly clustered, but are distributed throughout the genome ( Additional file 2: Figure S4).

The number of GHs possessed by *M. phaseolina* is higher than the average for plant pathogenic fungi and is nearly four times more abundant than the GTs, consistent with the importance of carbohydrate degradation rather than formation. There are 25 putative endoglucanases (GH5, GH12, GH45, GH61), 7 exocellobiohydrolases (GH6, GH7, GH81), and 28 β-glucosidases (GH1, GH3, GH17) for the hydrolysis of cellulose. The cellulolytic activity of *M. phaseolina* was shown to be significantly higher than that of other fungal species (i.e., *Aspergillus niger* and *Trichoderma reesei*) [26], reflecting the pathogenicity potency of this fungus.

The *M. phaseolina* genome also has the highest number of CEs than any other sequenced fungal genome so far (Table 4), with particular expansion of families CE9 (chitin metabolism) and CE10 (sterol esterases). Thirty two CE10 members are found in the *M. phaseolina* genome, but is absent in eight other species (Table 4). Nine CE5 candidate cutinases were found in the genome, suggesting that these enzymes are critical for initial penetration through the plant cuticle. The complement of pectin lyases (PL1, PL3, PL4), pectin hydrolases (GH28, GH88), and pectin esterases allows *M. phaseolina* to fully saccharify pectin. Other polysaccharide degrading enzymes predicted in the genome include catalytic activities for degrading starch and glycogen, hemicellulose, chitin, and β-glucans.

**Table 4:** **Comparison of the number of carbohydrate esterases of * M. phaseolina* with other fungi**

**Name**	**CE1**	**CE2**	**CE3**	**CE4**	**CE5**	**CE8**	**CE9**	**CE10**	**CE12**	**CE14**	**CE15**	**CE16**	**NC**
*C. neoformans* var. *neoformans*	2	0	0	4	0	0	1	0	0	0	0	0	0
*M. grisea*	10	1	6	8	15	1	1	0	2	0	1	1	1
*S. cerevisiae*	1	0	0	2	0	0	0	0	0	0	0	0	0
*P. anserina*	14	0	8	5	7	1	1	0	1	0	3	1	0
*A. nidulans*	3	0	6	7	4	3	1	0	2	0	0	3	4
*A. niger*	3	0	1	5	5	3	1	0	2	0	0	2	3
*A. oryzae*	5	0	3	3	5	5	1	0	4	0	0	3	1
*P. chrysogenum*	2	0	4	5	4	2	1	0	2	0	1	1	0
*M. phaseolina*	1	0	0	8	9	4	10	32	0	1	0	0	0

### Genes involved in lignin degradation

Major components of the lignin depolymerization system in *M. phaseolina* include laccases, lignin peroxidases, galactose oxidases, and chloroperoxidases, haloperoxidases, and heme peroxidases. *M. phaseolina* strain MS6 indeed demonstrates ligninolytic activity (Additional file 2: Figure S5). In comparison to seven other fungal species, *M. phaseolina* possesses the highest number of laccases (Table 5). Lignin peroxidase has been reported only in *Phanerochaete chrysosporium* thus far [27] and interestingly, this study revealed the second occurrence in *M. phaseolina*. Six extracellular class II heme peroxidases (IPR002016), 6 chloroperoxidases, and 7 haloperoxidases also contribute to the ligninolytic activity. In addition, we found a significant number of GMC oxidoreductases (40), which includes alcohol oxidases and cellobiose dehydrogenases. These enzymes are known to be directly involved in lignocellulosic degradation [27]. 

**Table 5:** **Comparison of the number of lignin degrading enzymes of * M. phaseolina* with other fungi**

**Fungal species**	**Laccase**	**Galactose oxidases**	**Lignin peroxidase**	**Chloroperoxidase**
*P. placenta*	2	0	0	5
*P. chrysosporium*	0	0	10	3
*C. neoformans*	0	0	0	0
*U. maydis*	0	1	0	0
*S. cerevisiae*	0	0	0	0
*A. nidulans*	1	0	0	0
*N. crassa*	5	1	0	0
*M. phaseolina*	22	7	3	6

### Virulence associated genes

As a wide host ranged pathogenic fungus, *M. phaseolina* is expected to possess a significant number of pathogen-host interaction genes. We searched the genome using the pathogen-host interaction database (PHI-base) [28] and identified 537 putative PHI genes (Additional file 1: Table S5). These genes play diverse roles in pathogenesis including adhesion, signal transduction, cell wall breakdown, purine biosynthesis, and biosynthesis of the potent mycotoxin patulin. ATP-binding cassette (ABC) transporters aid in defending the pathogen from host-produced secondary metabolites as well as provide essential nutrients [29]. A number of detoxification genes are present, such as those encoding cytochrome P450 (IPR001128), Cof protein (IPR000150), and superoxide dismutase, Cu/Zn binding (IPR001424). In addition, several beta-ketoacyl synthases (IPR000794) involved in the biosynthesis of a polyketide antibiotic [30] as well as some tetracycline resistance genes have been identified in *M. phaseolina.* These data suggest that the *M. phaseolina* genome encodes a large repertoire of pathogenicity-associated genes which may be involved in the pathogenesis of this organism.

### Signal transduction

The perception of environmental cues through cell-surface receptors and relaying the information to intracellular signaling pathways is essential for pathogenicity. The PTH11-like GPCR is a PHI protein shown to regulate *Magnaporthe grisea* appressorium differentiation in response to the plant surface [31]. The *M. phaseolina* genome has 44 putative PTH11-like GPCRs compared to an average of 34 in other pathogenic fungi (Table 2). The putative PTH11-like GPCRs are grouped into seven subfamilies. Three G-protein alpha subunits are present to transduce the extracellular signals leading to infection specific development, which is required for pathogenicity [32,33]. The 140 protein kinases in *M. phaseolina* is above the average (131) found in other ascomycete fungi (Table 2). Since signal transduction is a crucial part of fungal development and the infection process, indeed most of the kinases had orthologs in PHI-base (134/140). This result indicates that protein kinases in *M. phaseolina* might play a functional role in pathogen-host interaction.

### Transport and detoxification of compounds

Plant pathogenic fungi use a wide range of strategies to gain access to the carbon sources of their host plants and counter the plant defense response. The *M. phaseolina* genome encodes 839 transporter genes comprising 106 families ( Additional file 1: Table S6). Majority of the transporter genes (782/839) were similar to those cataloged in PHI-base. A large proportion of transporters belong to the MFS family (270), but the ABC superfamily (59) and Amino Acid-Polyamine-Organocation (APC) family (40) are also well represented in the genome ( Additional file 1: Table S6). *M. phaseolina* has more amino acid transporters (54) than other pathogenic fungi (29 to 38), revealing that this fungus might be able to access a wide range of protein degradation products from host sources. The sucrose and galactoside transporter (MFS superfamily) is required by *Metarhizium anisopliae* for rhizosphere competence but not for virulence [34]. The *M. phaseolina* genome has 15 sucrose and galactoside transporters, whereas *Fusarium graminearum* contains 12, suggesting these genes could be generally important for establishing plant-fungus relationships.

We found a relatively large number of genes involved in detoxification (Table 2). The dehydrogenases (411), acyl-CoA N-acetyltransferases (7), monooxygenases (104), and cytochrome P450s (256) were preferentially expanded in *M. phaseolina.* P450s play an important role in various hydroxylation and oxidation processes including secondary metabolism as well as the breakdown of toxins and other xenobiotic compounds [35]. For example, pisatin demethylase, a P450 from the plant pathogenic fungus *Nectria haematococca*, detoxifies a specific class of plant defense compounds [36]. The genome was particularly enriched in zinc-containing alcohol dehydrogenases (92) required for the biosynthesis of mannitol, a crucial factor for stress tolerance and virulence in the animal pathogen *Cryptococcus neoformans*[37]. The monooxygenases are generally involved in rapid elimination of plant polyphenols (which can act as antifungal agents), thus reducing the plant defense [38-40].

### Secondary metabolic pathways

Plant pathogenic fungi produce diverse secondary metabolites that aid in pathogenicity, such as host selective toxins [41]. We identified 75 putative secondary metabolite genes in the *M. phaseolina* genome, compared with 32 in *M. grisea*, 37 in *Botrytis cinerea*, 29 in *Sclerotinia sclerotiorum*, and 37 in *F. graminearum* (Table 6). There are 35 genes predicted to encode polyketide synthases (PKS), compared with 23 PKS genes in *M. grisea*. An impressive number of non-ribosomal peptide synthetases (NRPS) are found in *M. phaseolina*, which catalyze the production of cyclic peptides including numerous toxins. Only 6 NRPS genes and 8 hybrid PKS-NRPS are present in *M. grisea*, whereas 28 NRPS and 12 PKS-NRPS genes are in *M. phaseolina.* Virulence of several fungi (e.g., *Cochliobolus heterostrophus, C. miyabeanus, F. graminearum*, and *Alternaria brassicicola*) is mediated by particular siderophores, a class of secondary metabolites for iron uptake whose synthesis involves a NRPS [42]. *M. phaseolina* contains a NRPS (contig00285) which is similar to HTS1 (46% identity), the key enzyme responsible for the biosynthesis of the host-selective HC-toxin that confers the specificity of *Cochliobolus carbonum* to maize [43]. The NRPS-like proteins encoded by contig00324, contig00467, contig00109, contig00163, and contig00323 are most similar to Ace1, a PKS-NRPS hybrid that confers avirulence to *M. grisea* during rice infection [44]. 

**Table 6:** Distribution of secondary metabolite gene families in different plant pathogenic fungi

**Protein family**^**a**^	***M. phaseolina***	***M. grisea***	***B. cinerea***	***S. sclerotiorum***	***F. graminearum***
Secondary metabolite backbone genes	75	32	37	29	37
PKS	19	12	16	16	14
PKS like	16	3	6	2	1
NRPS	15	5	6	5	10
NRPS like	13	6	8	5	11
HYBRID	12	3	0	0	1
DMAT	0	3	1	1	0

^a^The abbreviations are *PKS*, polyketide synthase; *NRPS*, non-ribosomal peptide synthetase; *HYBRID*, hybrid PKS-NRPS enzyme; *DMAT*, dimethylallyl tryptophan synthase.

### Phenotypic response in relation to different environmental stimuli

**Figure 3:**
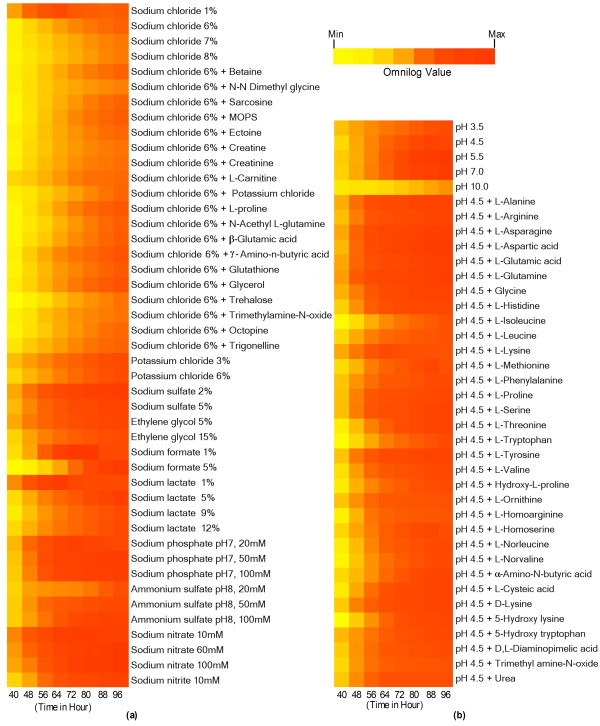
**Phenotype profiles of *M. phaseolina*. (a)** Osmotic/ion effects. **(b)** pH effects. Only conditions with a final OmniLog value (at 96 hr) ≥ 200 were incorporated into the heat maps. Color scale indicates the growth of the organism in particular substrate over time.

We used Phenotype Microarray (PM) analysis (Biolog Inc.) to evaluate *M. phaseolina* against ~960 different carbon, nitrogen, phosphorus, sulfur, nutrient supplement, peptide nitrogen, osmolytes, and pH sources (Figure 3; Additional file 2: Figures S6-S11). Of particular interest, we found that the adaptability to wide osmotic and pH ranges could be a contributing factor to this organism’s pervasive nature.

PM analysis revealed that *M. phaseolina* is capable of growing in sodium salt concentrations of 1-8%, with maximal growth at 2-4% (Figure 3a). In comparison, 3 % NaCl is toxic for *Saccharomyces cerevisiae* but is close to the optimum for growth of the halophilic black yeast *Hortaea werneckii*, one of the most salt-tolerant eukaryotic organisms so far described [45]. The efficient utilization of various osmolytes provides clues to *M. phaseolina*’s osmoadaptation strategy. It was recently shown that salinity increases the disease severity caused by *M. phaseolina* on *Phaseolus vulgaris* (common bean), by enhancing the growth rate of the pathogen as well as weakening the plant due to ion (K^+^ and Na^+^) imbalance [46]. This indicates that *M. phaseolina* could be a greater threat in areas with saline soils.

pH is one of the major environmental factors affecting pathogenicity. The *M. phaseolina* genome contains 2 putative PalH and 5 PalI proteins which are responsible for sensing ambient pH [47]. Moreover, there are several pH-regulated proteins including 19 acid phosphatases, 2 α-L-arabinofuranosidases, and 7 alkaline phosphatases. The presence of acid and alkaline phosphatases indicates that *M. phaseolina* has an extraordinary capability to neutralize both acidic and alkaline environments for its growth. This is clearly evident from our PM analysis. The results revealed that *M. phaseolina* can grow in pH ranging from strongly acidic to alkaline (pH 3.5 to 10), with maximum growth between pH 5 to 7 (Figure 3b). Therefore, *M. phaseolina* has a robust pH sensing system which enables it to adapt to adverse conditions.

### Pathogenesis of *M. phaseolina*

**Figure 4:**
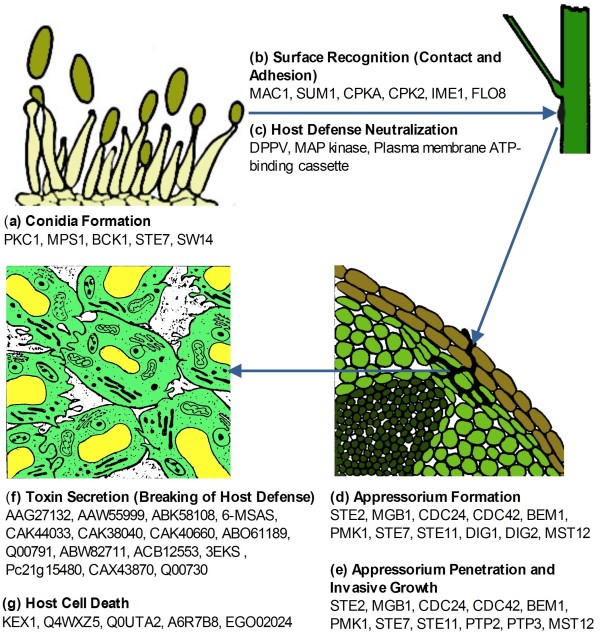
**Pathogenic lifestyle and infection process of *M. phaseolina.*** (**a**) Conidia or sclerotia are released from the pathogen. (**b**) Conidia or sclerotia disperse during early rainy season and contact the host tissue with the aid of transglutaminase-like proteins and cellulose-binding elicitor lectin. (**c**) Pathogen neutralizes the initial host defense with salicylate-1-monooxygenase. (**d**) Conidia form appressorium under the control of the central regulator PMK1. (**e**) Penetration peg invades into the plant epidermis. (**f**) Inside the host, the pathogen releases an array of different toxins and cell wall degrading enzymes and finally breakdown the host defense. (**g**) Results in host cell death and self-establishment.

The array of metabolic genes within the *M. phaseolina* genome reflects its pathogenic lifestyle (Figure 4; Additional file 1: Table S7).

**Figure 5:**
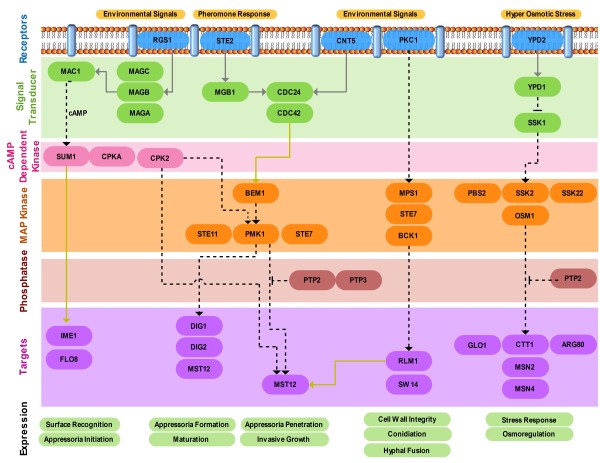
**Pathogenicity regulatory pathway of *M. phaseolina*.** Dotted line, Regulation; Solid line (gray), Molecular transport; Solid line (yellow-green), Protein modification.

The production and dispersal of conidia is important for fungal survival and infection of new hosts. Hyperosmotic stress is one of the environmental stimuli that often trigger conidiation [48]. The *M. phaseolina* genome encodes MPH_01444, a homolog of the MAP kinase OSM1, which regulates the osmotic stress response (to maintain cellular turgor) along with MPH_10325 and MPH_03305 (Figure 5; Additional file 1: Table S8). In *M. grisea*, deletion of *OSM1* has pleiotropic effects including osmotic sensitivity, reduced conidiation, and overproduction of appressoria [49]. 

Adhesion of fungal propagules to the plant surface is the prerequisite to establish disease. We identified 8 homologs of Cellulose-Binding Elicitor Lectin (CBEL), a cell surface glycoprotein that plays a role in adhesion to host wall components [50], as well as a Class II hydrophobic protein which mediates contact and communication between the fungus and its environment [51]. *M. phaseolina* also has three transglutaminase-like proteins containing a 13-amino acid motif (Pep-13) that is able to stimulate the plant defense response. Presence of these cell surface proteins suggests that *M. phaseolina* produces PAMPs, which can be efficiently perceived by a wide range of plant species. Activation of the plant immune system results in the synthesis of antifungal peptides, inhibitors of cell wall degrading enzymes, and phytoalexins. At the same time, the fungus responds to surface inductive cues via a cAMP-dependent pathway in order to initiate the infection process and combat the plant defense response (e.g., production of salicylate-1-monooxygenase).

Invasion begins with the emergence of a germ tube from the conidium, followed by appressorium formation. The developing appressorium swells as the cytoskeleton and Golgi vesicles accumulate in the tip. In bean rust pathogen *Uromyces appendiculatus*, the cytoskeleton and vesicles in the apex of the hypha are reorganized along the cell wall within 4 minutes of signal perception [52]. Enzymes for fungal cell wall synthesis (e.g., chitin synthase and β-1,3-glucan synthase) as well as cell wall degradation (e.g., cutinase and β-1,4-endoglucanase) are contained within different vesicles. *M. phaseolina* also synthesizes phytosphingosine and phytoceramide to protect the cell membrane from mechanical damage during penetration of the host cell. High turgor pressure is generated within the appressorium, allowing the penetration peg to break through the plant epidermis. Secretion of a variety of cell wall degrading enzymes and other toxins aid in the invasion of the host. These hyphae penetrate epidermal walls directly and subsequently colonize the tissue by intra- and intercellular growth (Figure 1c).

Regulation of these invasion processes involves both cAMP-dependent and MAP kinase pathways (Figure 5). Surface recognition and appressoria initiation is dependent on the adenylate cyclase *MAC1* (MPH_00975). Early research revealed that deletion of the *MAC1* gene blocked appressorium formation in *M. grisea*[53]. The catalytic subunit of cAMP-dependent protein kinase A (MPH_07566 and MPH_00397) is also required for appressoria formation and penetration. Furthermore, the *M. phaseolina* genome contains heterotrimeric G-proteins (MPH_02326, MPH_07066, MPH_00770, MPH_05860 and MPH_09815), which could also contribute to the signaling pathways for pathogenesis. The later stage of plant penetration is regulated by the MAP kinase *PMK1* (MPH_06596). A *PMK1* mutant of *M. grisea* is unable to make appressoria in rice plants and is non-pathogenic. Therefore, *PMK1* is specifically involved in the regulation of appressorium formation in response to surface signals and is also necessary for invasive growth [54].

## Conclusions

*M. phaseolina* is a soil-borne plant pathogenic fungus that infects major food, fiber, and oil crops, but it is also an opportunistic pathogen of humans [55]. Therefore, an understanding of the genetic basis underlying pathogenicity is crucial for controlling this pathogen.

Whole genome analysis showed that *M. phaseolina* is distinct from those of other known phytopathogenic fungi. We found 12% of the genes encoded by the genome have significant similarities with genes involved in pathogen-host interactions. *M. phaseolina* possesses a large repertoire of hydrolytic enzymes for degrading all major components of the plant cell wall and cuticle, including cellulose, hemicellulose, pectin, lignin, and cutin. The overall number of CAZymes is particularly high, primarily due to the abundance of GHs. Comparison with genomes of other phytopathogenic fungi reveals a distinct set of CEs in *M. phaseolina*, with significant expansion of families CE9 and CE10. CEs are considered as the first line of attack during host invasion and therefore play a key role in pathogenesis [56]. Extensive genetic diversity was also observed within complex gene families encoding peroxidases, oxidases, and cytochrome P450s. Interestingly, the genome has a considerable number of lignin peroxidases, extracellular Class II heme peroxidases, chloroperoxidases, and haloperoxidases compared with lignocellulose degrading fungus *P. chrysosporium*. The vast array of genes enables *M. phaseolina* to tackle nearly any type of cell wall composition it encounters, resulting in highly efficient penetration of different hosts and tissues.

We have identified differences in gene content among *M. phaseolina* and other plant pathogenic fungi. This is the first analysis of the genome of a plant pathogenic fungus that contains a large number of enzymes for the degradation of cell wall polysaccharides and lignocellulose. The *M. phaseolina* genome laid the foundation to elucidate its specialized mechanism to infect more than 500 plant hosts. Furthermore, it would decipher in-depth understanding of pathogenesis to resistance strategies.

## Methods

### Strain, growth condition, and nucleic acid isolation

*M. phaseolina* strain MS6 was isolated from an infected jute plant at Bangladesh Jute Research Institute (BJRI), Dhaka. Strain MS6 is the most virulent among the 19 isolates so far isolated in BJRI. This strain has mycelium that is grey-white at the initial stage and turns dark green at the mature stage. It is coarse with feathery strand. The sclerotia are embedded in the strand. The fungus was cultured at 30°C in liquid potato dextrose medium. DNA and RNA were extracted as previously described [57,58], respectively.

### Genome sequencing and assembly

Whole-genome shotgun sequencing of the *M. phaseolina* MS6 strain was performed using the 454 and Illumina sequencing platforms. We generated a total of 6.92 Gb raw data, having 40.56 millions of raw reads. In the 454 sequencing strategy, both single-end (SE) and paired-end (PE) genomic libraries were constructed, of which 2.38 Gb of shotgun sequences provided 48.29x coverage and 1.57 Gb of 8 kb, 15 kb, and 20 kb PE sequences provided 31.95x coverage of the *M. phaseolina* genome. In the Illumina sequencing strategy, 2.97 Gb of PE libraries were generated, of which 1.73 Gb of 500 bp PE sequences provided 35.11x coverage and 1.24 Gb of 3 kb mate-paired sequences provided 25.14x coverage of the *M. phaseolina* genome.

We produced a high quality assembly of the *M. phaseolina* genome using Newbler assembly program version 2.5.3 (http://my454.com/products/analysis-software/index.asp). For both *de novo* assembly and reference mapping of the *M. phaseolina* genome, we only used raw data generated from 454 pyrosequencing. For *de novo* assembly, we fed the Newbler GS *de novo* assembler first with the shotgun sequences in one-step form and later with paired-end sequences incrementally in order to get better contigging and scaffolding. About 96.50% raw reads were assembled into 3,036 contigs and 94 scaffolds having 98.92% bases with Q40 plus bases. For reference mapping, we used Newbler GS reference mapper to map the raw sequence files onto the all contigs file generated that gave 98.89% reads and 99.11% bases mapped to the reference.

We also used Illumina PE sequences with GapCloser version 1.10, a tool from SOAP *de novo* (http://soap.genomics.org.cn/soapdenovo.html), in order to close the gaps inside the scaffolds that were generated from the Newbler scaffolding process. A total of 785 gaps were detected by GapCloser that cover 1.5 Mb residues of which 197 gaps were completely filled up, leaving only 1.47% (0.73 Mb) gaps inside the 94 scaffolds.

We checked the relative completeness of the *M. phaseolina* MS6 draft assembly version 1.0 by performing core gene annotation using the CEGMA pipeline [59]. The resulting contigs as well as scaffolds from the *M. phaseolina* MS6 assembly were independently analyzed through this pipeline. In both cases we have found 245 (98.79%) complete gene models out of 248 ultra-conserved core eukaryotic genes (CEGs) present in the *M. phaseolina* genome.

This Whole Genome Shotgun project has been deposited at GenBank under the accession AHHD00000000. The version described in this paper is the first version, AHHD01000000.

### Genome annotation

We used Program to Assemble Spliced Alignments (PASA), a eukaryotic genome annotation pipeline [60], to generate potential training gene sets that were used to train other *ab initio* gene prediction software like Augustus v. 2.5.5 [61] and Glimmer HMM v. 3.0.1 [62] for predicting *M. phaseolina* genes.

A total of 13,481 gene assemblies under 11,414 gene clusters predicted by PASA, along with cDNA of *M. phaseolina* and other 4 closely related species (*Aspergillus nidulans*, *M. grisea*, *P. marneffei*, and *S. cerevisiae*) were used for this training purpose. Augustus and Glimmer independently predicted 12,231 and 11,432 ORFs, respectively, which were then subjected to correct gene structure annotation by EVidenceModeler (EVM) [63]. EVM, when combined with PASA, yields a comprehensive, configurable annotation system that predicts protein-coding genes and alternatively spliced isoforms.

We also used Analysis and Annotation Tool (AAT) [64] as a pipeline for transcript and protein alignments. To align the *M. phaseolina* genome independently, we used cDNA of its own with cDNA of other 4 related fungal species (described earlier) and fungal protein databases downloaded from Fungal Genome Research (http://fungalgenome.org/data/PEP/). These two along with PASA transcript alignments were used as transcript and protein evidences for EVM. Finally, a total of 14,249 *ab initios* were predicted of which 11,975 were corrected gene structures from EVM along with 2,274 genes from Augustus and Glimmer underlying the intergenic region of EVM predictions.

The *ab initios* were then subjected to InterProScan [65] and nr BLAST (threshold value of E < 10^-5^) [66] for searching functional domains and homologies. Results revealed a total of 10,250 genes with either potential domains or homologies with other fungal proteins and 3,999 novel genes out of 14,249 predicted protein coding genes of *M. phaseolina.*

Transfer RNA-coding regions were searched using tRNAscan-SE [67] and rRNA was searched using RNAmmer [68]. Repetitive elements were predicted by using RepeatMasker (http://www.repeatmasker.org/) and Putative Transposon elements were identified by Transposon-PSI (http://transposonpsi.sourceforge.net), a program that performs tBLASTn searches using a set of position specific scoring matrices (PSSMs) specific for different TE families.

The genomes of *M. phaseolina* and *F. oxysporum* were compared using MUMmer [69] tools to identify regions of synteny, with *Aspergillus fumigatus* used as a reference genome. For visualizing multiple genome comparisons, SyntenyMiner (http://syntenyminer.sourceforge.net/) was also used to visualize orthologous gene clusters among the organisms.

To identify proteins involved in carbohydrate metabolism, we used the Carbohydrate Active Enzymes (CAZy) database (http://www.cazy.org/). All CAZy related GenBank accession numbers were first downloaded from the CAZy website and then sequences were downloaded from NCBI using a custom python script. These sequences were searched by RPS-BLAST against the Pfam database to reveal protein domain architectures and compared against the Pfam domains identified in *M. phaseolina* proteins. The sequences were also compared by BLASTp against all *M. phaseolina* proteins to confirm the Pfam database matches.

Putative secondary metabolites (PKS and NRPS) were identified by using antiSMASH [70]. Pathogenicity and virulence associated genes were identified using the PHI-base database (http://www.phibase.org/), a database that catalogs experimentally verified pathogenicity, virulence, and effector genes from fungal, Oomycete, and bacterial pathogens which infect animal, plant, fungal and insect hosts. Briefly, all sequences were first downloaded from the PHI-base database and then compared by BLASTp against all *M. phaseolina* proteins to confirm the presence of homologous genes in the *M. phaseolina* genome.

The *in silico* predictions were manually curated and tested experimentally with several larger gene families such as CAZymes and lignin degrading protein coding genes by PCR.

### Construction of phylogenetic tree

Orthologous relationships were determined for a 14-way clustering from the complete genomes of the 14 fungal taxa: *Aspergillus nidulans, Fusarium oxysporum, Penicillium chrysogenum, Grosmannia clavigera, Magnaporthe grisea, Podospora anserina, M. phaseolina, Botryosphaeria dothidea, Laccaria bicolor, Phanerochaete chrysosporium, Postia placenta, Yarrowia lipolytica, Saccharomyces cerevisiae,* and *Trichoderma reesei*. All predicted protein sequences for the genomes of these fungi were searched against each other using BLASTp and clustered into orthologous groups using MCL-10-201. Single-copy orthologs were identified as the clusters with exactly one member per species. Phylogenetic relationships were determined from these single-copy orthologs and were aligned with MAFFT [71]. Alignments were pruned with Gblocks [72].

The evolutionary history was inferred by using the Maximum Likelihood method based on the JTT matrix-based model [73] and bootstrap value was 1,000. Evolutionary analyses were conducted in MEGA5 [74].

### Metabolic pathway reconstruction

The Yeast-5 Pathway Studio database (Ariadne Genomics) contains molecular interactions extracted by MedScan natural processing technology for all fungal species from over 1,000,000 Pubmed abstracts annotated with Medical Subject Headings (MeSH) term “Yeast OR Fungi” and from more than 100,000 full-length open access articles. Proteins in the Yeast-5 database are annotated with Entrez Gene and GenBank identifiers from six fungal genomes: *S. cerevisiae*, *Schizosaccharomyces pombe*, *Cryptococcus neoformans* var. *neoformans* JEC21, *A. fumigatus* Af293, A*. nidulans* FGSC A4, and *Aspergillus niger* CBS 513.88. To facilitate analysis of the *M. phaseolina* genome, we have added to the Yeast-5 database annotated proteins from recently sequenced genomes for *Ustilago maydis* 52 [75], *Metarhizium anisopliae* ARSEF 23, and *Metarhizium acridum* CQMa 102 [76].

The Yeast-5 database contains a collection of 303 metabolic pathways copied from MetaCyc. Pathways are represented as a collection of complex database entities called functional classes (enzymes) and a set of corresponding Chemical reactions. Every functional class in the database can contain an unlimited number of protein members performing corresponding enzymatic activity. Usually a set of members includes paralogs of catalytic and regulatory subunits necessary to perform enzymatic activity. The original Yeast-5 database has 444 functional classes with members out of a total of 769 pathways. We augmented the Yeast-5 database with an additional 168 metabolic pathways from RiceCyc and PoplarCyc to increase the pool of candidate enzymatic reactions for metabolic reconstruction of *M. phaseolina.*

The database of *M. phaseolina* interologs (predicted interactions) and reconstructed pathways was created by annotating proteins in the Yeast-5 database with *M. phaseolina* ortholog identifiers. Orthologs for *M. phaseolina* proteins in other fungal organisms were calculated using the best reciprocal hit method from full length protein sequence similarities calculated from BLAST alignments as described previously [77]. First, orthologs were calculated between *M. phaseolina* and each of the nine fungal genomes supported in the Yeast-5 database. The best ortholog was then chosen for each *M. phaseolina* protein among the nine possible ortholog pairs. All interactions extracted for *M. phaseolina* orthologs were exported from the Yeast-5 database along with pathways containing *M. phaseolina* orthologs*. M. phaseolina* interologs and predicted pathways were imported into a new Pathway Studio database for manual pathway reconstruction and genome analysis. Pathways that contained at least one functional class with no *M. phaseolina* orthologs were manually curated to achieve one of the following three outcomes: a) close the gap by finding members in the *M. phaseolina* genome and adding them to empty functional classes, b) dismiss entire pathway if gap cannot be closed, or c) remove enzymatic step if empty functional class represents redundant path in the pathway.

To identify paralog families in the *M. phaseolina* genome, we used BLASTP to calculate all possible protein homologs in the *M. phaseolina* genome and then selected only homologs that have 30% shared amino acid similarity calculated as the average sequence similarity between two homologs. Paralog pairs were imported into the *M. phaseolina* database as a new type of interaction called “Paralog”. Protein functional families were identified as clusters in the global Paralog network using the direct force layout algorithm. To assign biological function to each Paralog cluster we found Gene Ontology groups enriched by the proteins in the cluster or simply inspected available functional annotation for proteins in the cluster.

### Phenotype microarray analysis

Phenotype microarray (PM) is a high-throughput technique for screening the response of an organism against various substrates. *M. phaseolina* was evaluated using panels PM1 to PM10 (Biolog Inc.). The PM plates are denoted as PM1 and PM2A MicroPlates for Carbon sources; PM3B MicroPlate for Nitrogen sources; PM4A MicroPlate for Phosphorus and Sulfur sources; PM5 MicroPlate for Nutrient supplements; PM6, PM7, and PM8 MicroPlates for Peptide nitrogen sources; PM9 MicroPlate for Osmolytes; and PM10 MicroPlate for pH. There are 96 wells in each plate, so the substrate utilization patterns by the fungus were evaluated against a total of 960 substrates including 9 negative and 4 positive controls (see Additional file 3 for list of substrates).

*M. phaseolina* was grown on potato dextrose agar (PDA) at 30°C for 72 hr. Active hyphae was inoculated into 30 ml of liquid potato dextrose medium and incubated at 30°C for 60 hr. The mycelia from the liquid culture (~2 g) were washed with physiological buffer solution (10 mM sodium phosphate, pH 7.0, filter sterilized) at least 7 times to remove nutrient contamination. After washing, the mycelia were aliquoted into two 1.5 ml microcentrifuge tubes and macerated by a pellet pestle motor for 15 minutes. One ml of filamentous fungi inoculating fluid (FF-IF, Biolog) was added into each tube. The solution was transferred into a 15 ml falcon tube and 6 ml of FF-IF was added. The macerated mycelia were centrifuged at 3500 rpm for 5 minutes and the supernatant was discarded. Six to 8 ml of FF-IF was added to the tube and let stand for at least 40 minutes to settle down the bigger mycelial clumps. Approximately 2 ml from the clear upper portion was harvested for measuring the transmittance at OD_590nm._ The transmittance was adjusted to 62% for usable concentration of inoculums.

The inoculum suspensions along with different stock solutions were prepared as per the protocol standardized by Biolog (“PM Procedures for Filamentous Fungi”, 25-Aug-07). All the wells of PM 1–10 were inoculated with 100 μl of the inoculum suspensions and incubated in the OmniLog machine at 30°C for 96 hr. The instrument was programmed for recording data from each well in 15 minute intervals. After completion of incubation, the recorded data were extracted and analyzed using TIBCO Spotfire v23.0.0.320. The experiment was replicated three times.

It was observed that there was almost no growth in all the plates up to 40 hr of incubation. We therefore considered the 40 hr incubation readings as the baseline in our analysis. The data for each 8 hrs from three replications were averaged for analysis. Based on the 96 hr reading, all the figures (Figure 3; Additional file 2: Figures S6-S11) were constructed using OmniLog value ≥ 200.

### Verification of lignin degradation

We measured the ability of *M. phaseolina* to degrade lignin on modified Boyd and Kohlmeyer (B&K) agar medium containing 4 mM guaiacol along with 0.001% azure B dye [78]. The medium was inoculated with this fungus and incubated at 30°C in the dark. After 4 days of incubation, a halo of intense brownish white color was formed under and around the fungal colony, and the azure B dye turned from blue to white (Additional file 2: Figure S5). The growth of intense brownish white color fungal colony indicates a positive reaction resulting from guaiacol oxidation [79]. The disappearance of the blue colored medium is also evidence of peroxidase production ( Additional file 2: Figure S5). The discoloration of azure B dye has been positively correlated with the production of lignin peroxidase and Mn dependent peroxidase, but it does not indicate the presence of laccase [80].

## Abbreviations

AAT: Analysis and Annotation Tool; ABC: ATP-binding cassette; APC: Amino acid-polyamine-organocation; CAZy: Carbohydrate active enzyme; CBEL: Cellulose-binding elicitor lectin; CBM: Carbohydrate binding module; CE: Carbohydrate esterase; CEG: Core eukaryotic gene; DMAT: Dimethylallyl tryptophan synthase; EST: Expressed sequence tag; EVM: EVidenceModeler; GH: Glycoside hydrolase; GPCR: G-protein coupled receptor; GT: Glycosyltransferase; HYBRID: Hybrid PKS-NRPS enzyme; MeSH: Medical Subject Heading; MFS: Major facilitator superfamily; NCBI: National Center for Biotechnology Information; NRPS: Non-ribosomal peptide synthetase; ORF: Open reading frame; PAMP: Pathogen-associated molecular pattern; PASA: Program to Assemble Spliced Alignments; PDA: Potato dextrose agar; PE: Paired-end; PHI: Pathogen-host interaction; PKS: Polyketide synthase; PL: Polysaccharide lyase; PM: Phenotype microarray; PSSMs: Position specific scoring matrices; RIP: Repeat-induced point mutation.

## Competing interests

The authors declare that they have no competing interests.

## Authors’ contributions

MA designed the study; MSI, MSH, MMI, EME, AH, QMMH, MZH, BA, SR, MSR, MMA, SH, and XW performed the experiments; MSI, MSH, MMI, EME, AH, QMMH, MZH, BA, SR, MSR, MMA, and MA analyzed the data; MSI, MSH, JAS, and MA wrote the manuscript. All authors read and approved the final manuscript.

## Supplementary Material

Additional file 1 **Supplemental Table S1 to Table S8. **Table S1 provides the raw data generation statistics. Table S2 provides the assembly statistics of *M. phaseolina *genome. Table S3 provides the transcriptome assembly statistics. Table S4 lists the InterPro codes corresponding to the protein families in Table 2. Table S5 lists the virulence associated genes in *M. phaseolina*. Table S6 lists the transporter families in *M. phaseolina*. Table S7 details the *M. phaseolina* gene models corresponding to Figure 4. Table S8 details the *M. phaseolina* gene models corresponding to Figure 5. Click here for file

Additional file 2 **Supplemental Figure S1 to Figure S11, supporting data analyses. **Figure S1 indicates the number of unique and shared genes between *M. phaseolina* and *F. oxysporum*. Figure S2 shows the paralog network clusters of *M. phaseolina*. Figure S3 indicates the distribution of transposable elements over the genome. Figure S4 indicates the distribution of CAZymes against the 15 largest supercontigs of *M. phaseolina*. Figure S5 shows the positive guaiacol oxidation by *M. phaseolina* after 4 days of inoculation. Figures S6 to S11 show the PM profiles for various substrates. Click here for file

Additional file 3 **Supplemental methods. **This file provides the full list of substrates assayed in the PM experiments. Click here for file
