# Supplementary material for: Tools to kill: Genome of one of the most destructive plant pathogenic fungi Macrophomina phaseolina
Source: BMC Genomics. 2012 Sep 19;13:493. doi: 10.1186/1471-2164-13-493 (PMC3477038; doi:10.1186/1471-2164-13-493)
Supplement: Additional file 2 — Supplemental Figure S1 to Figure S11, supporting data analyses. Figure S1 indicates the number of unique and shared genes between M. phaseolina and F. oxysporum. Figure S2 shows the paralog network clusters of M. phaseolina. Figure S3 indicates the distribution of transposable elements over the genome. Figure S4 indicates the distribution of CAZymes against the 15 largest supercontigs of M. phaseolina. Figure S5 shows the positive guaiacol oxidation by M. phaseolina after 4 days of inoculation. Figures S6 to S11 show the PM profiles for various substrates. [file 1471-2164-13-493-S2.pdf]

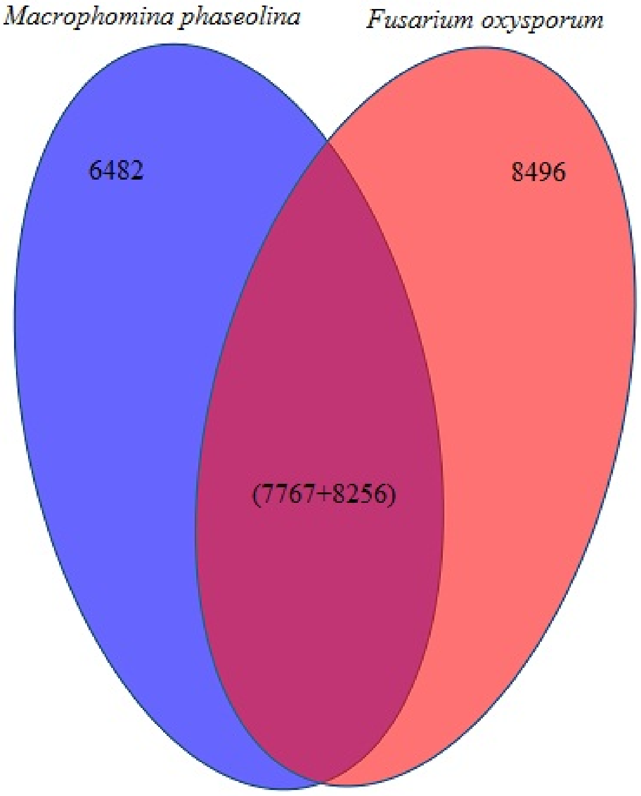

Figure S1. Venn diagram showing unique and shared genes between *M. phaseolina* and *F. oxysporum*

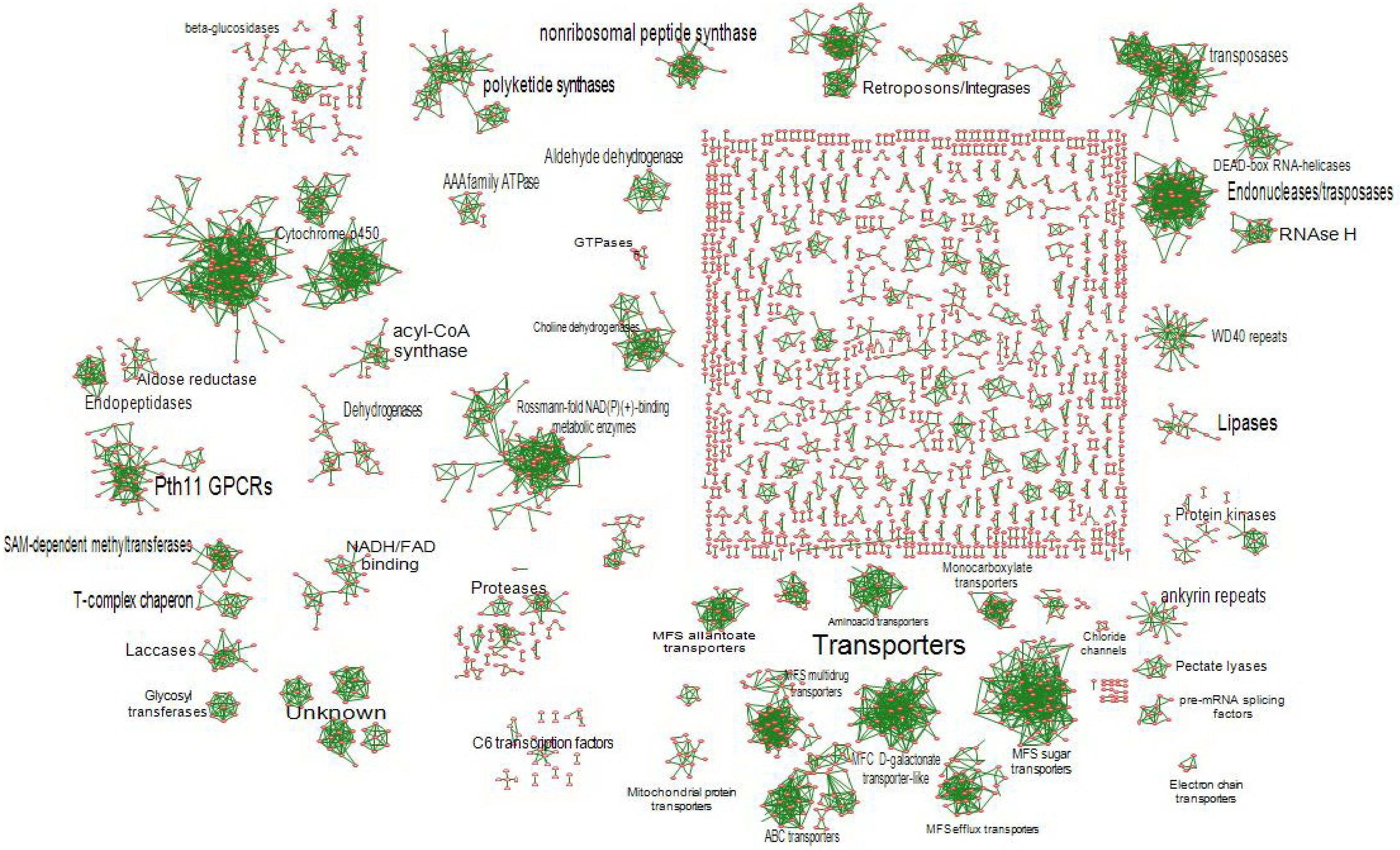

Figure S2. Functional protein families in the *M. phaseolina* genome identified as paralog network clusters (Pathway Studio)

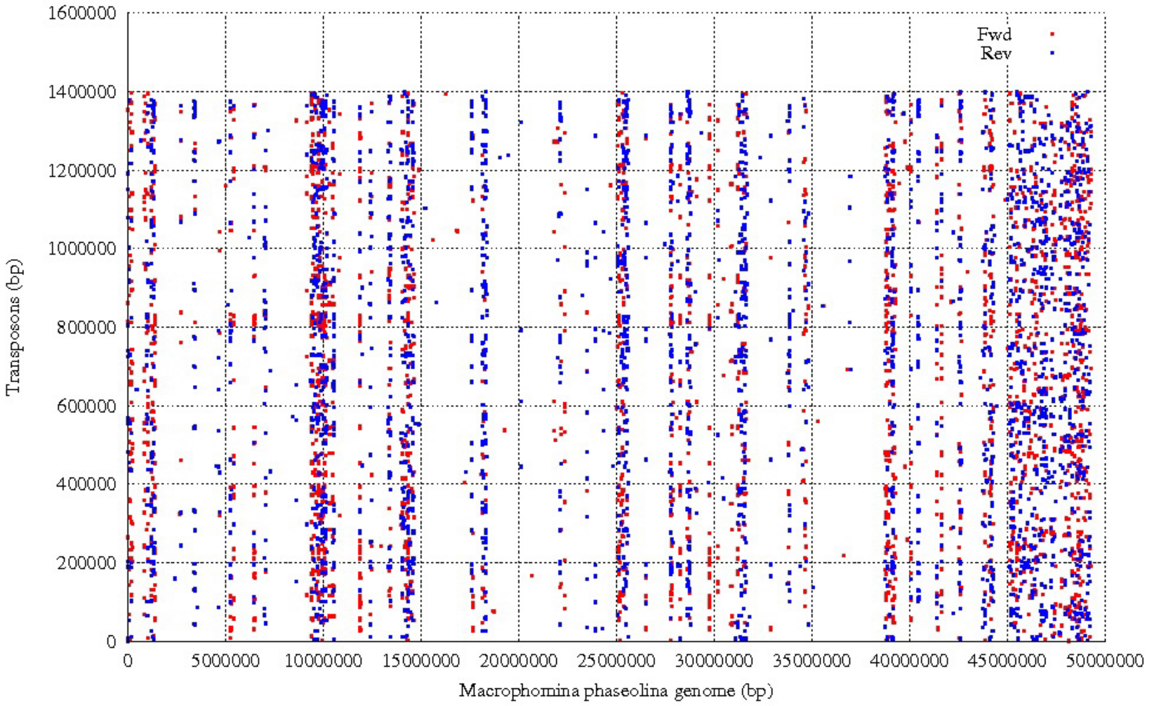

Figure S3. Distribution of transposable elements over the genome. All concatenated TEs were aligned with the *M. phaseolina* genome as concatenated contigs using MUMmer. The red and blue dots represent, corresponding, forwardly and reversely mapped TEs.

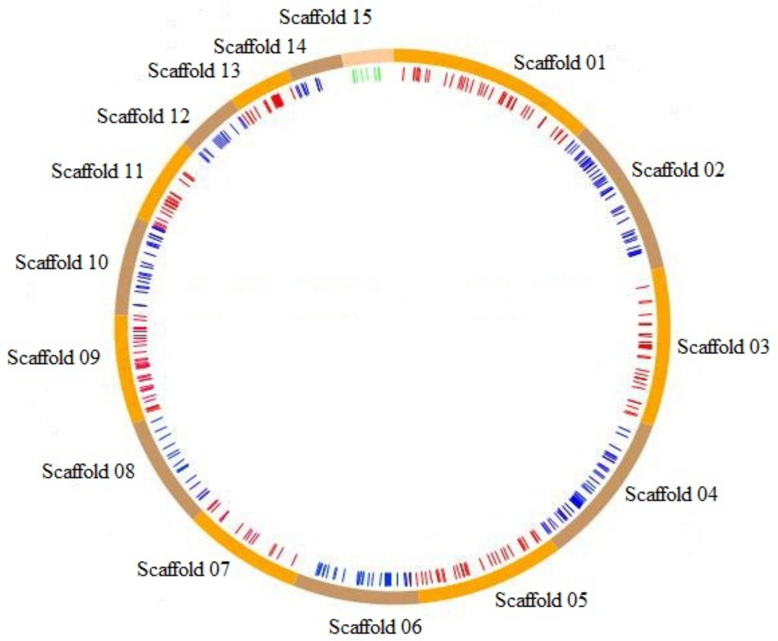

Figure S4. Distribution of CAZymes against the 15 largest supercontigs of *M. phaseolina*. Out of 362 CAZymes, 361 enzymes reside in these 15 supercontigs while one enzyme resides in supercontig 18. From scaffold 01 to scaffold 15, the orange, brown and light brown colors of the outer circle were used to differentiate the scaffolds. For the inner circle, the red, blue and green colors were used to differentiate the distribution of CAZymes among the 15 scaffolds.

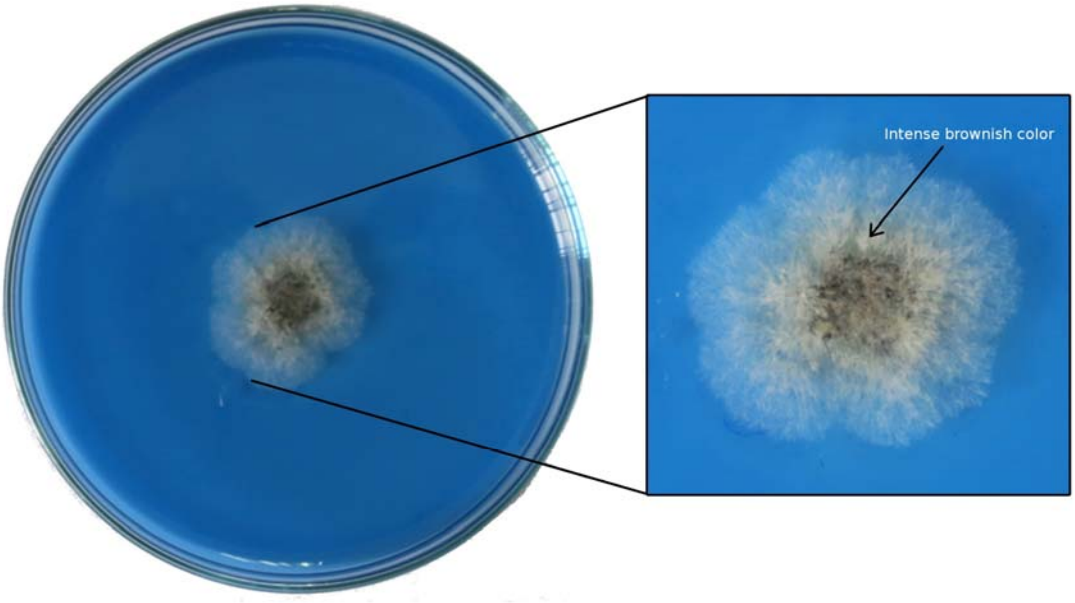

Figure S5. Photograph of modified B&K agar plate containing 4 mM guaiacol with azure B dye showing positive guaiacol oxidation as well as discoloration of azure B dye by *M. phaseolina* after 4 days of inoculation. This is an evidence of ligninolytic activity.

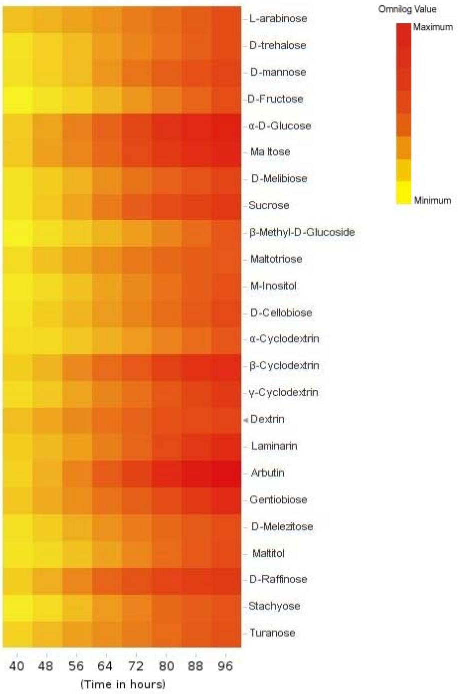

Figure S6. Substrate utilization profile of *M. phaseolina* in different carbon sources. Only conditions with a final OmniLog value (at 96 hr) ≥ 200 were incorporated into the heat map. Color scale indicates the growth of the organism in particular substrate over time.

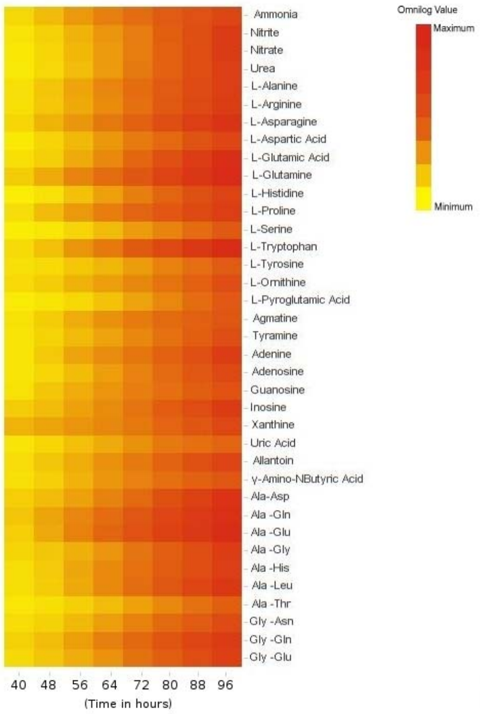

Figure S7. Substrate utilization profile of *M. phaseolina* in different nitrogen sources.

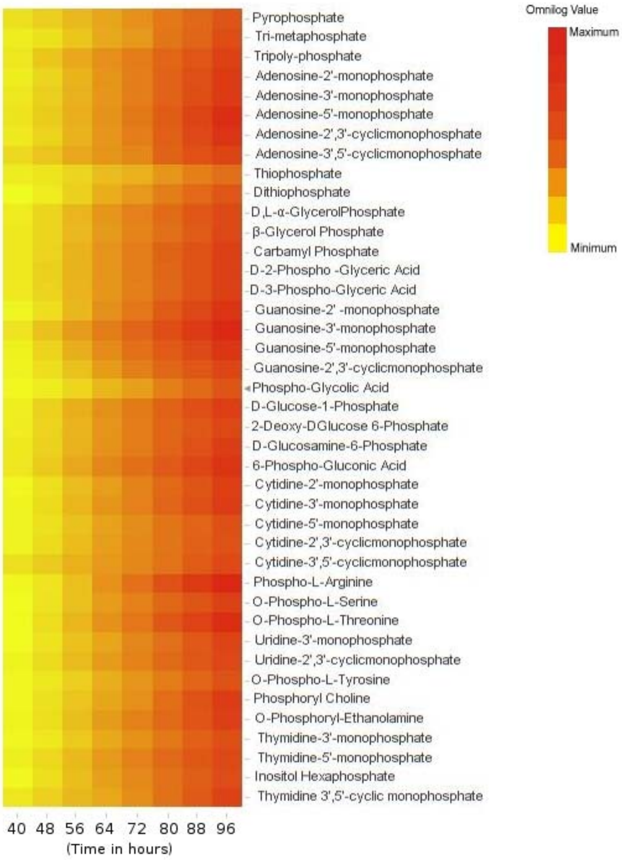

Figure S8. Substrate utilization profile of *M. phaseolina* in different phosphorus sources

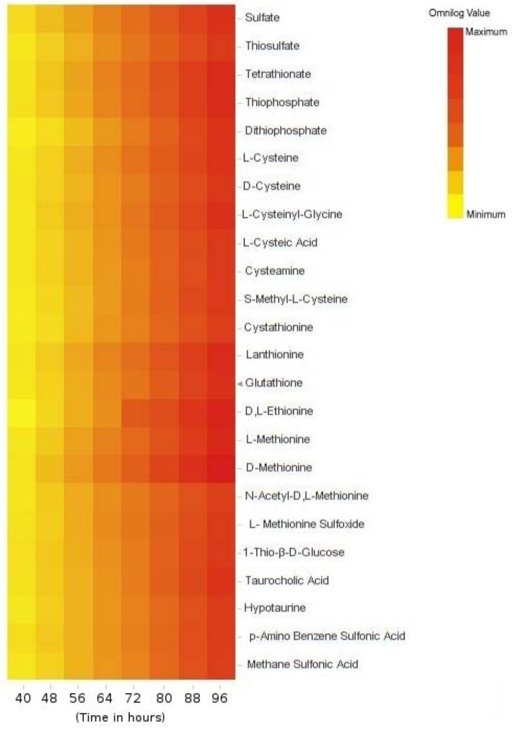

Figure S9. Substrate utilization profile of *M. phaseolina* in different sulfur sources

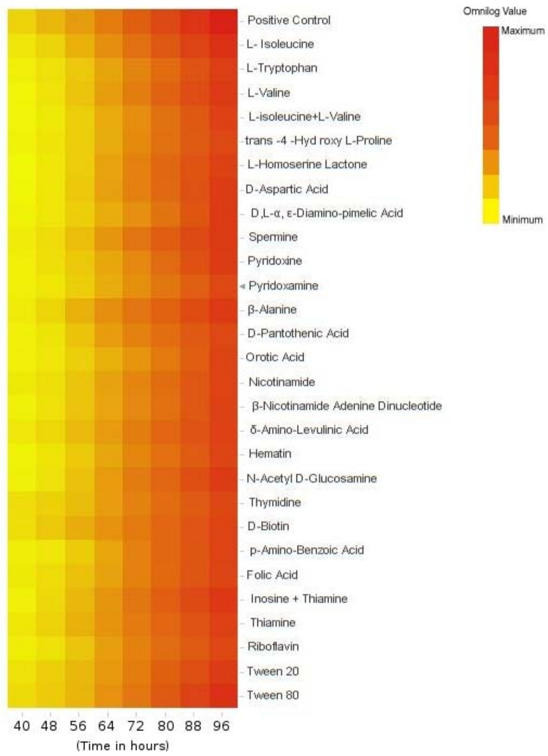

Figure S10. Substrate utilization profile of *M. phaseolina* in different nutrient supplements

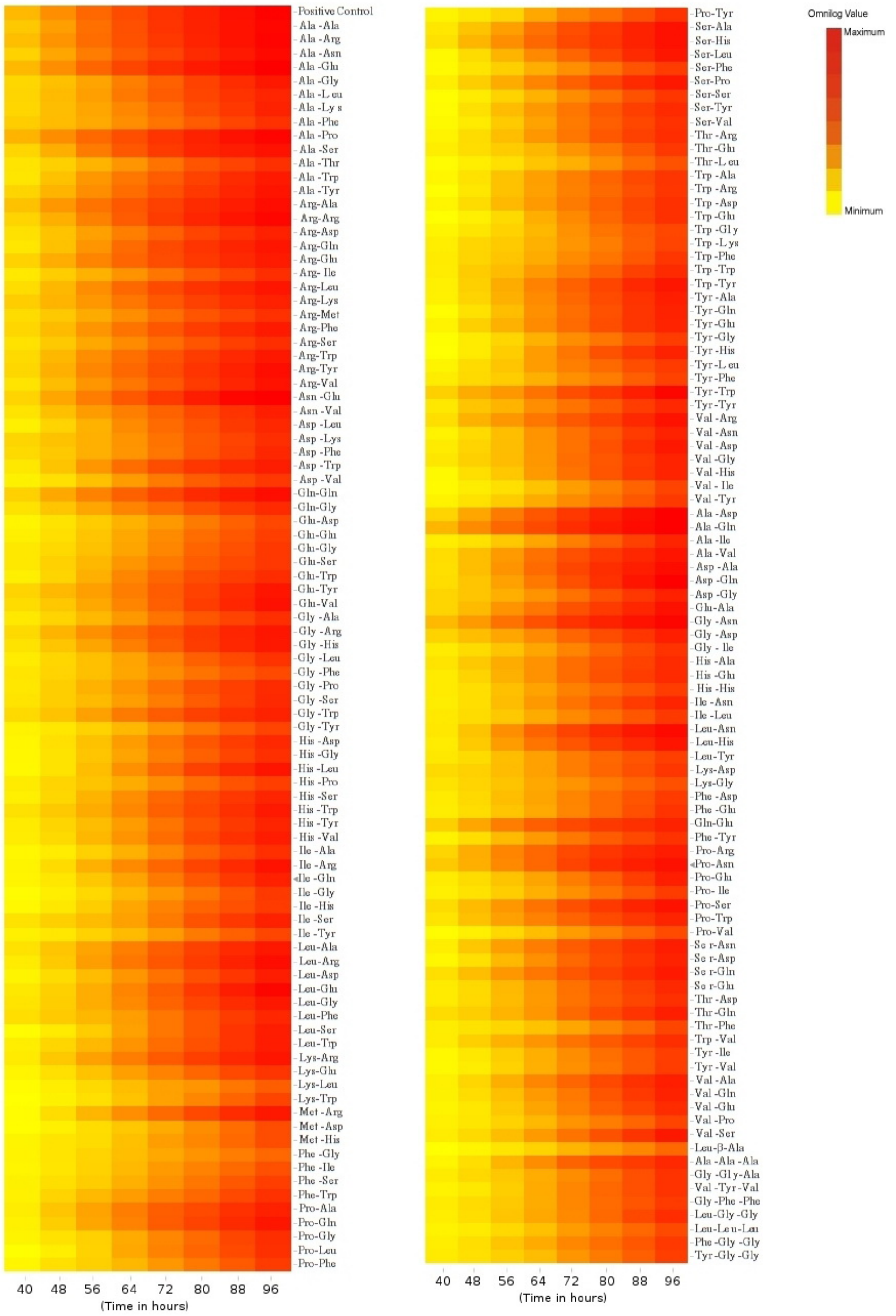

Figure S11. Substrate utilization profile of *M. phaseolina* in different peptide nitrogen sources
