# Supplementary material for: Tools to kill: Genome of one of the most destructive plant pathogenic fungi Macrophomina phaseolina
Source: BMC Genomics. 2012 Sep 19;13:493. doi: 10.1186/1471-2164-13-493 (PMC3477038; doi:10.1186/1471-2164-13-493)
Supplement: Additional file 3 — Supplemental methods. This file provides the full list of substrates assayed in the PM experiments. [file 1471-2164-13-493-S3.doc]

**Additional Data File 3:** **Supplemental methods**

**List of substrates used in Phenotype Microarray experiments**

**PM1 MicroPlate for Carbon sources**

Water (negative control), L-arabinose, N-acetyl-D-glucosamine, D-saccharic acid, Succinic acid, D-galactose, L-aspartic acid, L-proline, D-alanine, D-trehalose, D-mannose, Dulcitol, D-serine, D-sorbitol, Glycerol, L-fucose, D-glucuronic acid, D-gluconic acid, D,L-α-glycerol phosphate, D-xylose, L-lactic acid, Formic acid, D-mannitol, L-glutamic acid, D-glucose-6-phosphate, D-galactonic acid-γ-lactone, D,L-Malic acid, D-Ribose, Tween 20, L-Rhamnose, D-Fructose, Acetic Acid, α-D-Glucose, Maltose, D-Melibiose, Thymidine, L-Asparagine, D-Aspartic Acid, D-Glucosaminic Acid, 1,2-Propanediol, Tween 40, α-Ketoglutaric acid, α-Ketobutyric acid, α-Methyl-D-Galactoside, α-D-Lactose, Lactulose, Sucrose, Uridine, L-Glutamine, m-Tartaric Acid, D-Glucose-1-Phosphate, D-Fructose-6-Phosphate, Tween 80, α-Hydroxyglutaric Acid-γ-Lactone, α-Hydroxybutyric Acid, β-Methyl-D-Glucoside, Adonitol, Maltotriose, 2-Deoxyadenosine, Adenosine, Gly-Asp, Citric Acid, m-Inositol, D-Threonine, Fumaric Acid, Bromosuccinic acid, Propionic Acid, Mucic Acid, Glycolic Acid, Glyoxylic Acid, D-Cellobiose, Inosine, Gly-Glu, Tricarballylic acid, L-Serine, L-Threonine, L-Alanine, Ala-Gly, Acetoacetic Acid, N-Acetyl-β-D-Mannosamine, Mono-Methylsuccinate, Methylpyruvate, D-Malic Acid, L-Malic Acid, Gly-Pro, p-Hydroxyphenyl Acetic acid, m-Hydroxyphenyl Acetic acid, Tyramine, D-Psicose, L-Lyxose, Glucuronamide, Pyruvic Acid, L-Galactonic Acid-γ-Lactone, D-Galacturonic acid, Phenylethylamine, 2-Aminoethanol

**PM2A MicroPlate for Carbon sources**

Negative control, Chondroitin Sulfate C, α-Cyclodextrin, β-Cyclodextrin, γ-Cyclodextrin, Dextrin, Gelatin, Glycogen, Inulin, Laminarin, Mannan, Pectin, N-Acetyl-D-Galactosamine, N-Acetyl Neuraminic acid, β-D-Allose, Amygdalin, D-Arabinose, D-Arabitol, L-Arabitol, Arbutin, 2-Deoxy-D-Ribose, i-Erythritol, D-Fucose, 3-O-β-D-Galactopyranosyl-D-Arabinose, Gentiobiose, L-Glucose, Lactitol, D-Melezitose, Maltitol, α-Methyl-D-Glucoside, β-Methyl-D-Galactoside, 3-Methyl Glucose, β-Methyl-D-Glucuronic Acid, α-Methyl-D-Mannoside, β-Methyl-D-Xyloside, Palatinose, D-Raffinose, Salicin, Sedoheptulosan, L-Sorbose, Stachyose, D-Tagatose, Turanose, Xylitol, N-Acetyl-D-Glucosaminitol, γ-Amino Butyric Acid, δ-Amino Valeric Acid, Butyric Acid, Capric Acid, Caproic Acid, Citraconic Acid, D,L-Citramalic Acid, D-Glucosamine, 2-Hydroxybenzoic Acid, 4-Hydroxybenzoic Acid, β-Hydroxybutyric Acid, γ-Hydroxybutyric Acid, α-Keto Valeric Acid, Itaconic Acid, 5-Keto-D-Gluconic Acid, D-Lactic Acid Methyl Ester, Malonic Acid, Melibonic Acid, Oxalic Acid, Oxalomalic Acid, Quinic Acid, D-Ribono-1,4-Lactone, Sebacic Acid, Sorbic Acid, Succinamic Acid, D-Tartaric Acid, L-Tartaric Acid, Acetamide, L-Alaninamide, N-Acetyl-L-glutamic acid, L-Arginine, Glycine, L-Histidine, L-Homoserine, Hydroxy-L-Proline, L-Isoleucine, L-Leucine, L-Lysine, L-Methionine, L-Ornithine, L-Phenylalanine, L-Pyroglutamic Acid, L-Valine, D,L-Carnitine, sec-Butylamine, D,L-Octopamine, Putrescine, Dihydroxyacetone, 2,3-Butanediol, 2,3-Butanone, 3-Hydroxy-2-Butanone

**PM3B MicroPlate for Nitrogen sources**

Negative control, Ammonia, Nitrite, Nitrate, Urea, Biuret, L-Alanine, L-Arginine, L-Asparagine, L-Aspartic Acid, L-Cysteine, L-Glutamic Acid, L-Glutamine, Glycine, L-Histidine, L-Isoleucine, L-Leucine, L-Lysine, L-Methionine, L-Phenylalanine, L-Proline, L-Serine, L-Threonine, L-Tryptophan, L-Tyrosine, L-Valine, D-Alanine, D-Asparagine, D-Aspartic Acid, D-Glutamic Acid, D-Lysine, D-Serine, D-Valine, L-Citrulline, L-Homoserine, L-Ornithine, N-Acetyl-D,L-Glutamic Acid, N-Phthaloyl-L-glutamic Acid, L-Pyroglutamic Acid, Hydroxylamine, Methylamine, N-Amylamine, N-Butylamine, Ethylamine, Ethanolamine, Ethylenediamine, Putrescine, Agmatine, Histamine, β-Phenylethylamine, Tyramine, Acetamide, Formamide, Glucuronamide, D,L-Lactamide, D-Glucosamine, D-Galactosamine, D-Mannosamine, N-Acetyl-D-Glucosamine, N-Acetyl-Galactosamine, N-Acetyl-D-Mannosamine, Adenine, Adenosine, Cytidine, Cytosine, Guanine, Guanosine, Thymine, Thymidine, Uracil, Uridine, Inosine, Xanthine, Xanthosine, Uric Acid, Alloxan, Allantoin, Parabanic Acid, D,L-α-Amino-N-Butyric Acid, γ-Amino-N-Butyric Acid, ε-Amino-N-Caproic Acid, D,L-α-Amino-Caprylic Acid, δ-Amino-N-Valeric Acid, α-Amino-N-Valeric Acid, Ala-Asp, Ala-Gln, Ala-Glu, Ala-Gly, Ala-His, Ala-Leu, Ala-Thr, Gly-Asn, Gly-Gln, Gly-Glu, Gly-Met, Met-Ala

**PM4A MicroPlate for Phosphorus**

Negative Control, Phosphate, Pyrophosphate, Trimetaphosphate, Tripolyphosphate, Triethyl Phosphate, Hypophosphite, Adenosine-2’-monophosphate, Adenosine-3’-monophosphate, Adenosine-5’-monophosphate, Adenosine-2’,3’-cyclic monophosphate, Adenosine-3’,5’-cyclic monophosphate, Thiophosphate, Dithiophosphate, D,L-α-Glycerol Phosphate, β-Glycerol Phosphate, Carbamyl Phosphate, D-2-Phospho-Glyceric Acid, D-3-Phospho-Glyceric Acid, Guanosine-2’–monophosphate, Guanosine-3’-monophosphate, Guanosine-5’-monophosphate, Guanosine-2’,3’-cyclic monophosphate, Guanosine-3’,5’-cyclic monophosphate, Phosphoenol Pyruvate, Phospho-Glycolic Acid, D-Glucose-1-Phosphate, D-Glucose-6-Phosphate, 2-Deoxy-D-Glucose 6-Phosphate, D-Glucosamine-6-Phosphate, 6-Phospho-Gluconic Acid, Cytidine-2’-monophosphate, Cytidine-3’-monophosphate, Cytidine-5’-monophosphate, Cytidine-2’,3’-cyclic mono-phosphate, Cytidine-3’,5’-cyclic monophosphate, D-Mannose-1-Phosphate, D-Mannose-6-Phosphate, Cysteamine-S-Phosphate, Phospho-L-Arginine, O-Phospho-D-Serine, O-Phospho-L-Serine, O-Phospho-L-Threonine, Uridine-2’-monophosphate, Uridine-3’-monophosphate, Uridine-5’-monophosphate, Uridine-2’,3’-cyclic monophosphate, Uridine-3’,5’-cyclic monophosphate, O-Phospho-D-Tyrosine, O-Phospho-L-Tyrosine, Phosphocreatine, Phosphoryl Choline, O-Phosphoryl-Ethanolamine, Phosphono Acetic Acid, 2-Aminoethyl Phosphonic Acid, Methylene Diphosphonic Acid, Thymidine-3’-monophosphate, Thymidine-5’-monophosphate, Inositol Hexaphosphate, Thymidine 3’,5’-cyclic monophosphate

**For Sulfur**

Negative Control, Sulfate, Thiosulfate, Tetrathionate, Thiophosphate, Dithiophosphate, L-Cysteine, D-Cysteine, Cys-Gly, L-Cysteic Acid, Cysteamine, L-Cysteine Sulfinic Acid, N-Acetyl-L-Cysteine, S-Methyl-L-Cysteine, Cystathionine, Lanthionine, Glutathione, D,L-Ethionine, L-Methionine, D-Methionine, Gly-Met, N-Acetyl-D,L-Methionine, L-Methionine Sulfoxide, L-Methionine Sulfone, L-Djenkolic acid, Thiourea, 1-Thio-β-D-Glucose, D,L-Lipoamide, Taurocholic Acid, Taurine, Hypotaurine, p-Aminobenzene Sulfonic Acid, Butane Sulfonic Acid, 2-Hydroxyethane Sulfonic Acid, Methane Sulfonic Acid, Tetramethylene Sulfone

**PM5 MicroPlate for Nutrient Supplements**

Negative Control, Positive Control, L-Alanine, L-Arginine, L-Asparagine, L-Aspartic Acid, L-Cysteine, L-Glutamic Acid, Adenosine-3’,5’-cyclic monophosphate, Adenine, Adenosine, 2’-Deoxyadenosine, L-Glutamine, Glycine, L-Histidine, L-Isoleucine, L-Leucine, L-Lysine, L-Methionine, L-Phenylalanine, Guanosine-3’,5’-cyclic monophosphate, Guanine, Guanosine, 2’-Deoxyguanosine, L-Proline, L-Serine, L-Threonine, L-Tryptophan, L-Tyrosine, L-Valine, L-isoleucine + L-Valine, trans-4-Hydroxy L-Proline, (5)4-Amino-Imidazole-4(5)-Carboxamide, Hypoxanthine, Inosine, 2’-Deoxyinosine, L-Ornithine, L-Citrulline, Chorismic Acid, (-)Shikimic Acid, L-Homoserine Lactone, D-Alanine, D-Aspartic Acid, D-Glutamic Acid, D,L-α,ε-Diamino-pimelic Acid, Cytosine, Cytidine, 2’-Deoxycytidine, Putrescine, Spermidine, Spermine, Pyridoxine, Pyridoxal, Pyridoxamine, β-Alanine, D-Pantothenic Acid, Orotic Acid, Uracil, Uridine, 2’-Deoxyuridine, Quinolinic Acid, Nicotinic Acid, Nicotinamide, β-Nicotinamide Adenine Dinucleotide, δ-Amino-Levulinic Acid, Hematin, Deferoxamine Mesylate, D-(+)-Glucose, N-Acetyl-D-Glucosamine, Thymine, Glutathione (reduced form), Thymidine, Oxaloacetic Acid, D-Biotin, Cyano-Cobalamine, p-Amino-Benzoic Acid, Folic Acid, Inosine + Thiamine, Thiamine, Thiamine Pyrophosphate, Riboflavin, Pyrrolo-Quinoline Quinone, Menadione, m-Inositol, Butyric Acid, D,L-α-Hydroxybutyric Acid, α-Ketobutyric Acid, Caprylic Acid, D,L-Thioctic acid, D,L-Mevalonic Acid lactone, D,L-Carnitine, Choline, Tween 20, Tween 40, Tween 60, Tween 80

**PM6 MicroPlate for Peptide Nitrogen Sources**

Negative Control, Positive Control : L-Glutamine, Ala–Ala, Ala–Arg, Ala–Asn, Ala–Glu, Ala–Gly, Ala–His, Ala-Leu, Ala-Lys, Ala–Phe, Ala–Pro, Ala–Ser, Ala–Thr, Ala–Trp, Ala–Tyr, Arg-Ala, Arg-Arg, Arg-Asp, Arg-Gln, Arg-Glu, Arg-Ile, Arg-Leu, Arg-Lys, Arg-Met, Arg-Phe, Arg-Ser, Arg-Trp, Arg-Tyr, Arg-Val, Asn–Glu, Asn–Val, Asp–Asp, Asp–Glu, Asp–Leu, Asp–Lys, Asp–Phe, Asp–Trp, Asp–Val, Cys–Gly, Gln-Gln, Gln-Gly, Glu-Asp, Glu-Glu, Glu-Gly, Glu-Ser, Glu-Trp, Glu-Tyr, Glu-Val, Gly–Ala, Gly–Arg, Gly–Cys, Gly–Gly, Gly–His, Gly–Leu, Gly–Lys, Gly–Met, Gly–Phe, Gly–Pro, Gly–Ser, Gly–Thr, Gly–Trp, Gly–Tyr, Gly–Val, His–Asp, His–Gly, His–Leu, His–Lys, His–Met, His–Pro, His–Ser, His–Trp, His–Tyr, His–Val, Ile–Ala, Ile–Arg, Ile–Gln, Ile–Gly, Ile–His, Ile–Ile, Ile–Met, Ile–Phe, Ile–Pro, Ile–Ser, lle–Trp, Ile–Tyr, Ile–Val, Leu-Ala, Leu-Arg, Leu-Asp, Leu-Glu, Leu-Gly, Leu-Ile, Leu-Leu, Leu-Met, Leu-Phe

**PM7 MicroPlate for Peptide Nitrogen Sources**

Negative Control, Positive Control: L-Glutamine, Leu-Ser, Leu-Trp, Leu-Val, Lys-Ala, Lys-Arg, Lys-Glu, Lys-Ile, Lys-Leu, Lys-Lys, Lys-Phe, Lys-Pro, Lys-Ser, Lys-Thr, Lys-Trp, Lys-Tyr, Lys-Val, Met–Arg, Met–Asp, Met–Gln, Met–Glu, Met–Gly, Met–His, Met –Ile, Met–Leu, Met–Lys, Met–Met, Met–Phe, Met–Pro, Met–Trp, Met–Val, Phe–Ala, Phe –Gly, Phe–Ile, Phe–Phe, Phe–Pro, Phe–Ser, Phe-Trp, Pro-Ala, Pro-Asp, Pro-Gln, Pro-Gly, Pro-Hyp, Pro-Leu, Pro-Phe, Pro-Pro, Pro-Tyr, Ser-Ala, Ser-Gly, Ser-His, Ser-Leu, Ser-Met, Ser-Phe, Ser-Pro, Ser-Ser, Ser-Tyr, Ser-Val, Thr–Ala, Thr–Arg, Thr-Glu, Thr-Gly, Thr-Leu, Thr–Met, Thr-Pro, Trp–Ala, Trp–Arg, Trp–Asp, Trp–Glu, Trp-Gly, Trp-Leu, Trp-Lys, Trp–Phe, Trp-Ser, Trp–Trp, Trp–Tyr, Tyr–Ala, Tyr–Gln, Tyr–Glu, Tyr–Gly, Tyr–His, Tyr-Leu, Tyr-Lys, Tyr–Phe, Tyr–Trp, Tyr–Tyr, Val–Arg, Val–Asn, Val–Asp, Val–Gly, Val–His, Val–Ile, Val–Leu, Val–Tyr, Val–Val, γ-Glu–Gly

**PM8 MicroPlate for Peptide Nitrogen Sources**

Negative Control, Positive Control : L-Glutamine, Ala–Asp, Ala–Gln, Ala–lle, Ala-Met, Ala–Val, Asp–Ala, Asp–Gln, Asp–Gly, Glu-Ala, Gly–Asn, Gly–Asp, Gly–lle, His–Ala, His–Glu, His–His, Ile–Asn, Ile–Leu, Leu-Asn, Leu-His, Leu-Pro, Leu-Tyr, Lys-Asp, Lys-Gly, Lys-Met, Met–Thr, Met–Tyr, Phe–Asp, Phe–Glu, Gln-Glu, Phe–Met, Phe–Tyr, Phe–Val, Pro-Arg, Pro-Asn, Pro-Glu, Pro-Ile, Pro-Lys, Pro-Ser, Pro-Trp, Pro-Val, Ser-Asn, Ser-Asp, Ser-Gln, Ser-Glu, Thr–Asp, Thr–Gln, Thr–Phe, Thr–Ser, Trp–Val, Tyr–lle, Tyr–Val, Val–Ala, Val–Gln, Val–Glu, Val–Lys, Val–Met, Val–Phe, Val-Pro, Val–Ser, β-Ala-Ala, β-Ala–Gly, β-Ala–His, Met-β-Ala, β-Ala–Phe, D-Ala-D-Ala, D-Ala-Gly, D-Ala-Leu, D-Leu-D-Leu, D-Leu-Gly, D-Leu-Tyr, γ-Glu–Gly, γ-D-Glu–Gly, Gly-D-Ala, Gly-D-Asp, Gly-D-Ser, Gly-D-Thr, Gly-D-Val, Leu-β-Ala, Leu-D-Leu, Phe-β–Ala, Ala-Ala–Ala, D-Ala-Gly–Gly, Gly-Gly-Ala, Gly-Gly-D-Leu, Gly-Gly-Gly, Gly-Gly-lle, Gly-Gly-Leu, Gly-Gly-Phe, Val-Tyr–Val, Gly-Phe–Phe, Leu-Gly–Gly, Leu-Leu-Leu, Phe-Gly–Gly, Tyr-Gly–Gly

**PM9 MicroPlate for Osmolytes**

1% NaCl, 2% NaCl, 3% NaCl, 4% NaCl, 5% NaCl, 5.5% NaCl, 6% NaCl, 6.5% NaCl, 7% NaCl, 8% NaCl, 9% NaCl, 10% NaCl, 6% NaCl, 6% NaCl + Betaine, 6% NaCl + N,N Dimethyl glycine, 6% NaCl + Sarcosine, 6% NaCl + Dimethyl sulphonyl propionate, 6% NaCl + MOPS, 6% NaCl + Ectoine, 6% NaCl + Choline, 6% NaCl + Phosphorylcholine, 6% NaCl + Creatine, 6% NaCl + Creatinine, 6% NaCl + L-Carnitine, 6% NaCl + KCl, 6% NaCl + L-proline, 6% NaCl + N-Acethyl-L-glutamine, 6% NaCl + β-Glutamic acid, 6% NaCl + γ–Amino-N-butyric acid, 6% NaCl + Glutathione, 6% NaCl + Glycerol, 6% NaCl + Trehalose, 6% NaCl + Trimethylamine-N-oxide, 6% NaCl + Trimethylamine, 6% NaCl + Octopine, 6% NaCl + Trigonelline, 3% Potassium chloride, 4% Potassium chloride, 5% Potassium chloride, 6% Potassium chloride, 2% Sodium sulfate, 3% Sodium sulfate, 4% Sodium sulfate, 5% Sodium sulfate, 5% Ethyleneglycol, 10% Ethyleneglycol, 15% Ethyleneglycol, 20% Ethyleneglycol, 1% Sodium formate, 2% Sodium formate, 3% Sodium formate, 4% Sodium formate, 5% Sodium formate, 6% Sodium formate, 2% Urea, 3% Urea, 4% Urea, 5% Urea, 6% Urea, 7% Urea, 1% Sodium Lactate, 2% Sodium Lactate, 3% Sodium Lactate, 4% Sodium Lactate, 5% Sodium Lactate, 6% Sodium Lactate, 7% Sodium Lactate, 8% Sodium Lactate, 9% Sodium Lactate, 10% Sodium Lactate, 11% Sodium Lactate, 12% Sodium Lactate, 20mM Sodium Phosphate pH 7, 50mM Sodium Phosphate pH 7, 100mM Sodium Phosphate pH 7, 200mM Sodium Phosphate pH 7, 20mM Sodium Benzoate pH 5.2, 50mM Sodium Benzoate pH 5.2, 100mM Sodium Benzoate pH 5.2, 200mM Sodium Benzoate pH 5.2, 10mM Ammonium sulfate pH 8, 20mM Ammonium sulfate pH 8, 50mM Ammonium sulfate pH 8, 100mM Ammonium sulfate pH 8, 10mM Sodium Nitrate, 20mM Sodium Nitrate, 40mM Sodium Nitrate, 60mM Sodium Nitrate, 80mM Sodium Nitrate, 100mM Sodium Nitrate, 10mM Sodium Nitrite, 20mM Sodium Nitrite, 40mM Sodium Nitrite, 60mM Sodium Nitrite, 80mM Sodium Nitrite, 100mM Sodium Nitrite

**PM10 MicroPlate for pH**

pH 3.5, pH 4, pH 4.5, pH 5, pH 5.5, pH 6, pH 7, pH 8, pH 8.5, pH 9, pH 9.5, pH 10, pH 4.5, pH 4.5 + L-Alanine, pH 4.5 + L-Arginine, pH 4.5 + L-Asparagine, pH 4.5 + L-Aspartic Acid, pH 4.5 + L-Glutamic Acid, pH 4.5 + L-Glutamine, pH 4.5 + Glycine, pH 4.5 + L-Histidine, pH 4.5 + L- Isoleucine, pH 4.5 + L-Leucine, pH 4.5 + L-Lysine, pH 4.5 + L-Methionine, pH 4.5 + L-Phenylalanine, pH 4.5 + L-Proline, pH 4.5 + L-Serine, pH 4.5 + L-Threonine, pH 4.5 + L-Tryptophan, pH 4.5 + L-Tyrosine, pH 4.5 + L-Valine, pH 4.5 + Hydroxy-L-Proline, pH 4.5 + L-Ornithine, pH 4.5 + L-Homoarginine, pH 4.5 + L-Homoserine, pH 4.5 + Anthranilic acid, pH 4.5 + L-Norleucine, pH 4.5 + L-Norvaline, pH 4.5 + α-Amino-N-butyric acid, pH 4.5 + p-Aminobenzoate, pH 4.5 + L-Cysteic acid, pH 4.5 + D-Lysine, pH 4.5 + 5-Hydroxy Lysine, pH 4.5 + 5-Hydroxy Tryptophan, pH 4.5 + D,L-Diaminopimelic acid, pH 4.5 + Trimethylamine-N-oxide, pH 4.5 + Urea, pH 9.5, pH 9.5 + L-Alanine, pH 9.5 + L-Arginine, pH 9.5 + L-Asparagine, pH 9.5 + L-Aspartic Acid, pH 9.5 + L-Glutamic Acid, pH 9.5 + L-Glutamine, pH 9.5 + Glycine, pH 9.5 + L-Histidine, pH 9.5 + L-Isoleucine, pH 9.5 + L-Leucine, pH 9.5 + L-Lysine, pH 9.5 + L-Methionine, pH 9.5 + L-Phenylalanine, pH 9.5 + L-Proline, pH 9.5 + L-Serine, pH 9.5 + L-Threonine, pH 9.5 + L-Tryptophan, pH 9.5 + L-Tyrosine, pH 9.5 + L-Valine, pH 9.5 + Hydroxy-L-Proline, pH 9.5 + L-Ornithine, pH 9.5 + L-Homoarginine, pH 9.5 + L-Homoserine, pH 9.5 + Anthranilic acid, pH 9.5 + L-Norleucine, pH 9.5 + L-Norvaline, pH 9.5 + Agmatine, pH 9.5 + Cadaverine, pH 9.5 + Putrescine, pH 9.5 + Histamine, pH 9.5 + Phenylethylamine, pH 9.5 + Tyramine, pH 9.5 + Creatine, pH 9.5 + Trimethylamine-N-oxide, pH 9.5 + Urea, X-Caprylate, X–α-D-Glucoside, X-β-D-Glucoside, X-α-D-Galactoside, X-β-D-Galactoside, X-α-D-Glucuronide, X-β-D-Glucuronide, X-β-D-Glucosaminide, X-β-D-Galactosaminide, X-α-D-Mannoside, X-PO4, X-SO4
